# Integrating High-Content Imaging and Chemical Genetics to Probe Host Cellular Pathways Critical for *Yersinia Pestis* Infection

**DOI:** 10.1371/journal.pone.0055167

**Published:** 2013-01-30

**Authors:** Krishna P. Kota, Brett Eaton, Douglas Lane, Melanie Ulrich, Ricky Ulrich, Brian D. Peyser, Camenzind G. Robinson, James G. Jaissle, Gianluca Pegoraro, Sina Bavari, Rekha G. Panchal

**Affiliations:** 1 Department of Target Discovery and Cellular Microbiology, United States Army Medical Research Institute of Infectious Diseases, Frederick, Maryland, United States of America; 2 Perkin Elmer, Waltham, Massachusetts, United States of America; 3 Target Structure Based Drug Discovery Group, SAIC-Frederick, NCI-Frederick, Frederick, Maryland, United States of America; 4 Pathology Division, United States Army Medical Research Institute of Infectious Diseases, Frederick, Maryland, United States of America; 5 Diagnostic Systems Division, United States Army Medical Research Institute of Infectious Diseases, Frederick, Maryland, United States of America; The Scripps Research Institute and Sorrento Therapeutics, Inc., United States of America

## Abstract

The molecular machinery that regulates the entry and survival of *Yersinia pestis* in host macrophages is poorly understood. Here, we report the development of automated high-content imaging assays to quantitate the internalization of virulent *Y. pestis* CO92 by macrophages and the subsequent activation of host NF-κB. Implementation of these assays in a focused chemical screen identified kinase inhibitors that inhibited both of these processes. Rac-2-ethoxy-3 octadecanamido-1-propylphosphocholine (a protein Kinase C inhibitor), wortmannin (a PI3K inhibitor), and parthenolide (an IκB kinase inhibitor), inhibited pathogen-induced NF-κB activation and reduced bacterial entry and survival within macrophages. Parthenolide inhibited NF-κB activation in response to stimulation with Pam3CSK4 (a TLR2 agonist), *E. coli* LPS (a TLR4 agonist) or *Y. pestis* infection, while the PI3K and PKC inhibitors were selective only for *Y. pestis* infection. Together, our results suggest that phagocytosis is the major stimulus for NF-κB activation in response to *Y. pestis* infection, and that *Y. pestis* entry into macrophages may involve the participation of protein kinases such as PI3K and PKC. More importantly, the automated image-based screening platform described here can be applied to the study of other bacteria in general and, in combination with chemical genetic screening, can be used to identify host cell functions facilitating the identification of novel antibacterial therapeutics.

## Introduction

The bacterial invasion of mammalian cells is often visualized directly by microscopy or quantitated by flow cytometry using fluorescently labeled bacteria [1]. Intracellular bacterial survival and replication can be determined by plating lysates from infected cells and enumerating colony counts, although this is a time consuming and tedious technique. Modulation of host responses to bacterial infection are often studied using global approaches such as microarrays or semi-quantitative low-throughput biochemical methods such as Western blots. Traditional genetic-based methods have also been applied to identify bacterial and host cell-specific factors that are required for intracellular pathogenesis. While all these approaches provide significant insight into the biology of disease pathogenesis, they are not amenable for the high-throughput screening of small molecules or siRNAs to identify antibacterial compounds or cellular targets that are involved in host-pathogen interactions.

Advanced automated high-throughput, high-content imaging (HCI) technologies are widely exploited in a variety of settings ranging from cellular signaling pathway analysis to drug-discovery [2]–[4]. In particular, HCI offers a valuable platform to profile phenotypic responses to molecular or pharmacologic perturbations at the cellular level, thereby enabling the further characterization of novel, unpredicted mechanisms of drug action - irrespective of the primary target [5]–[7]. In conjunction with techniques for multi-parametric clustering analysis, many cellular features related to texture, shape, size, fluorescence intensity, and localization can be extracted and analyzed from complex heterogeneous cell populations [8]–[11]. Image-based phenotypic screens have recently been adapted for functional genomic studies and to discover novel antimicrobials for intracellular bacterial pathogens such as *Chlamydia*, *Salmonella* and *Mycobacteria* [12]–[15].

In this study, we developed high-throughput automated imaging assays to quantitate bacterial phagocytosis and activation of the host NF-κB signaling pathway during the infection of macrophages by virulent *Yersinia pestis* CO92, a Gram-negative facultative pathogen that is the causative agent of plague [16]. To probe host cell functions and factors involved in macrophage and *Y. pestis* interactions, we screened a library containing 1280 pharmacologically active small molecules in both of the optimized assays. Several compounds that inhibit *Y. pestis* phagocytosis and/or NF-κB signaling within macrophages were identified. The majority of the identified bioactive small molecule hits from both assays were kinase inhibitors. Our results suggest the involvement of protein kinases such as PKC and PI3K during *Y. pestis* internalization.

## Results

### Imaging Phagocytosis and Enumerating Internalized Bacteria

**Figure 1:**
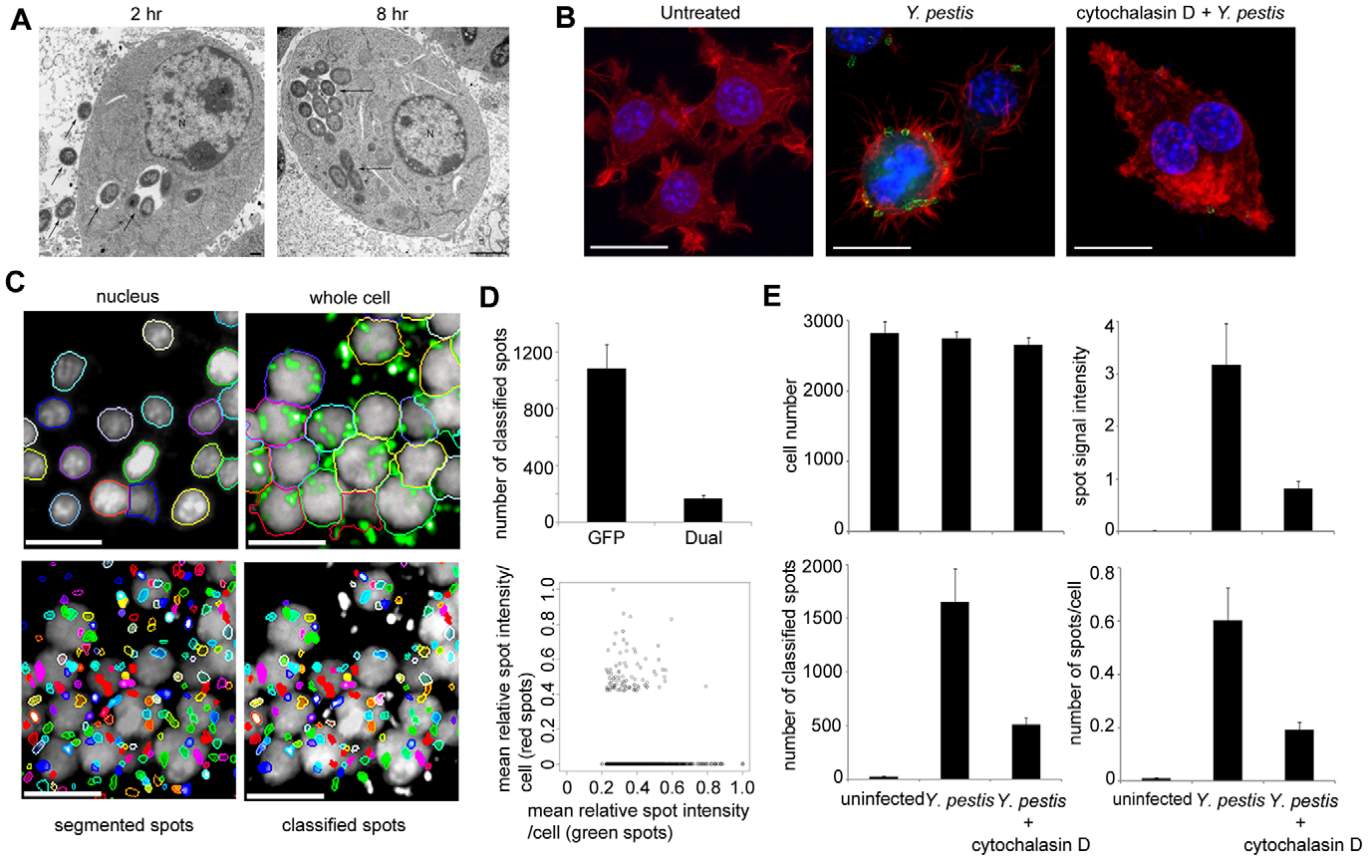
Quantitative imaging of internalized *Y. pestis*. (**A**) Transmission electron microscopy showing *Y. pestis* CO92 internalized within RAW264.7 macrophages at 2 hr (left panel; scale bar –500 nm) and 8 hr (right panel; scale bar –2 microns) post infection. Some extracellular bacteria (arrow outside the cell) are also visible at 2 hr post infection. Bacteria confined within the vacuoles of the macrophages are shown by a single arrow inside the cell. N - Nucleus. (**B**) Immunofluorescent staining of RAW264.7 macrophages that were not infected (left panel) or infected with *Y*. *pestis* CO92 following pre-treatment with DMSO control (middle panel) or cytochalasin D (right panel). Green - *Y*. *pestis* stained with α-F1 antibody, red - phalloidin, blue – nucleus stained with Hoechst dye. Scale bar = 5 µm. (**C**) Image segmentation of macrophages infected with *Y. pestis*. Images acquired in UV channel were segmented to mark the boundaries of the nuclei (top left), cell boundaries were segmented on the images based on a 640 nm laser channel (cytoplasm channel, top right), segmentation of total spots (bacteria) for image fields acquired in the 488 nm channel (bottom left), classification of spot candidates (internalized bacteria) based on the attribute values for contrast and spot-to-cell intensity above the limits set by the input parameters (bottom right). The different colored spots in the bottom panels represent individual spots (bacteria) and were used for better visualization. The non-segmented white spots in the bottom right panel represent extracellular bacteria. Scale bar = 20 µm. (**D**) Dual staining of GFP expressing *Y. pestis* (Pgm^−^, Pla^−^) in infected RAW264.7 macrophages to quantitate cell-associated and internalized bacteria. RAW264.7 macrophages were infected for 2 hr with 30∶1 MOI of GFP expressing bacteria. After fixation but without permeabilization of the macrophages, the GFP labeled bacteria were immunostained with αF1 antibody followed by staining with Alexa 568 secondary antibody. Graphical representation of number of bacteria expressing GFP alone or dual staining is shown in the Top panel. Bottom panel is the scatter plot representation of single cell analysis of triplicate wells. The mean relative spot (bacteria) intensity in the red channel is plotted against the green channel for all cells with ≥ one classified spot. (**E**) Enumerating internalized bacteria. Graphical representations of the four output features (see Table S1) that were collected from the image analysis of RAW264.7 macrophages that were either not infected or infected with *Y*. *pestis* following pretreatment with DMSO control or cytochalasin D. The data represents the average of six replicates ± standard deviation, and is representative of three independent experiments.

*Y. pestis* exhibits a dual intracellular and extracellular lifestyle. During infection of RAW264.7, a murine macrophage-like cell line (referred throughout as RAW264.7 macrophages), a fraction of the *Y. pestis* are observed to be intracellular, which we confirmed by electron microscopy studies. As shown in **Figure 1A**, intracellular *Y. pestis* CO92 were observed to be confined within vacuoles of macrophages at 2 hr (left panel) and at 8 hr (right panel) post-infection. Next, we set up a high-throughput, cell-based imaging assay to quantitate internalized bacteria. RAW264.7 macrophages were infected with the virulent *Y. pestis* CO92 at multiplicity of infection (MOI) of 30∶1. After incubation for 2 hr, cells were washed, fixed and permeabilized. The bacteria were detected using an F1 monoclonal primary antibody [17] that binds to the associated capsule antigen present on the surface of *Y. pestis* and Dylight 488 secondary antibody. An automated high-throughput confocal fluorescent microscope was then used to acquire high-resolution, single plane images of infected macrophages and fluorescently labeled bacteria (**Figure 1B**). Optimal conditions for phagocytosis were first determined using an avirulent strain of *Y*. *pestis* (Pgm^−^, Pst^−^). RAW264.7 macrophages infected with this strain showed a time and MOI dependent increase in uptake of the bacteria that could be quantitatively measured using an image analysis script (**Figure S1**). These optimized conditions were then applied to the evaluation of the pathogenic *Y. pestis* CO92 (**Figure 1B**).

To discriminate extracellular bacteria from the cell-associated and internalized bacteria, confocal Z stack images were acquired at different Z planes which confirmed the internalized *Y. pestis* within RAW264.7 macrophages, although some cell-associated external bacteria were also observed (**Movie S1**). To quantitate the percentage of cell-associated and internalized *Y. pestis*, an automated image analysis script was used to segment the cell nuclei (**Figure 1C** **, top left**) and the cytoplasm (**Figure 1C** **, top right**) and to count the foci of fluorescently labeled *Y. pestis* present in the cellular mask (**Figure 1C** **, bottom panels**). To further validate our image analysis script to discriminate cell-membrane associated bacteria from internalized bacteria, a *Y. pestis* CO92 derivative (Pgm^−^, Pla^−^) expressing GFP in trans was used to infect RAW264.7 macrophages. After fixation but without permeabilization of the macrophages, the GFP labeled bacteria were immunostained with α F1 antibody followed by staining with Alexa 568 secondary antibody. Image analysis revealed that while a majority of the bacteria were intracellular (corresponding to the spots displaying a fluorescent signal only in the GFP channel), about 15% of the GFP positive bacteria were cell-associated and not internalized as they exhibited dual staining in the GFP and Alexa 568 channels (**Figure 1D** **Top panel)**. These results were further confirmed following single cell analysis of triplicate wells and by quantitating the mean relative spot (bacteria) signal intensity per cell in the Alexa 568 (red) and GFP (green) channels (**Figure 1D**, **bottom panel**). Spots of varying fluorescent intensity in the GFP channel were observed depending on the location of the bacteria above or below the focal plane. In summary these results indicated that the image analysis algorithm used in these studies can accurately discriminate and quantitate extracellular bacteria versus cell-associated and internalized bacteria.

During image processing, several features were captured as described in **Table S1** and analyzed (**Figure 1E**). The top four features that best describe phagocytosis were: i) the number of classified spots, which is proportional to the number of cell-associated and internalized bacteria; ii) integrated spot signal intensity, which is representative of the total fluorescence intensity associated with the antibody-labeled bacteria. This parameter is important to determine if the bacteria are clumping, which may cause inaccurate segmentation of the spots (*i.e* bacteria); iii) total number of macrophages, which is a very useful indicator of cell loss caused either by compound toxicity and/or bacterial infection and; iv) average number of bacterial spots per cell, which is calculated based on the total number of macrophages present per well. This parameter becomes important when there is cell loss resulting from compound or pathogen cytotoxicity. For example, cell loss might lead to reduced bacterial spot count, thus generating false positive results during compound screening.

Phagocytosis of *Yersinia* by macrophages and non-phagocytic cells is an actin-dependent process [18], [19]. To evaluate the importance of host cell actin rearrangement on *Y. pestis* uptake and to confirm that our high-content assay is capable of quantitatively measuring the phagocytosis process, RAW264.7 macrophages were pretreated with cytochalasin D, an inhibitor of actin polymerization [20], and then infected with *Y. pestis* CO92 (**Figure 1B, right panel**). The number of fluorescent foci corresponding to the internalized bacteria was measured. A 3–5 fold reduction in cellular entry of the bacteria was observed in macrophages pretreated with cytochalasin D and infected with *Y. pestis* compared to the infection control (**Figure 1E**). Subsequent statistical evaluation of the HCI assay indicated that the assay is robust, with a Z’ value of 0.5, thus validating its potential application for small molecule inhibitor screening.

### Screening of Pharmacologically Active Compounds in the Phagocytosis Assay

To evaluate the effects of small molecules on modulating the process of *Y. pestis* phagocytosis, the Sigma-Aldrich LOPAC^1280^ library containing pharmacologically active compounds (as well as FDA approved drugs) was screened at a single concentration (20 µM) in the optimized phagocytosis assay. For each plate, values were normalized to the median of all infected wells in the plate using a robust Z-score. Principal components analysis was then applied to integrate the multiple output features extracted during image analysis relating to the internalization of *Y*. *pestis* by RAW264.7 macrophages.

**Figure 2:**
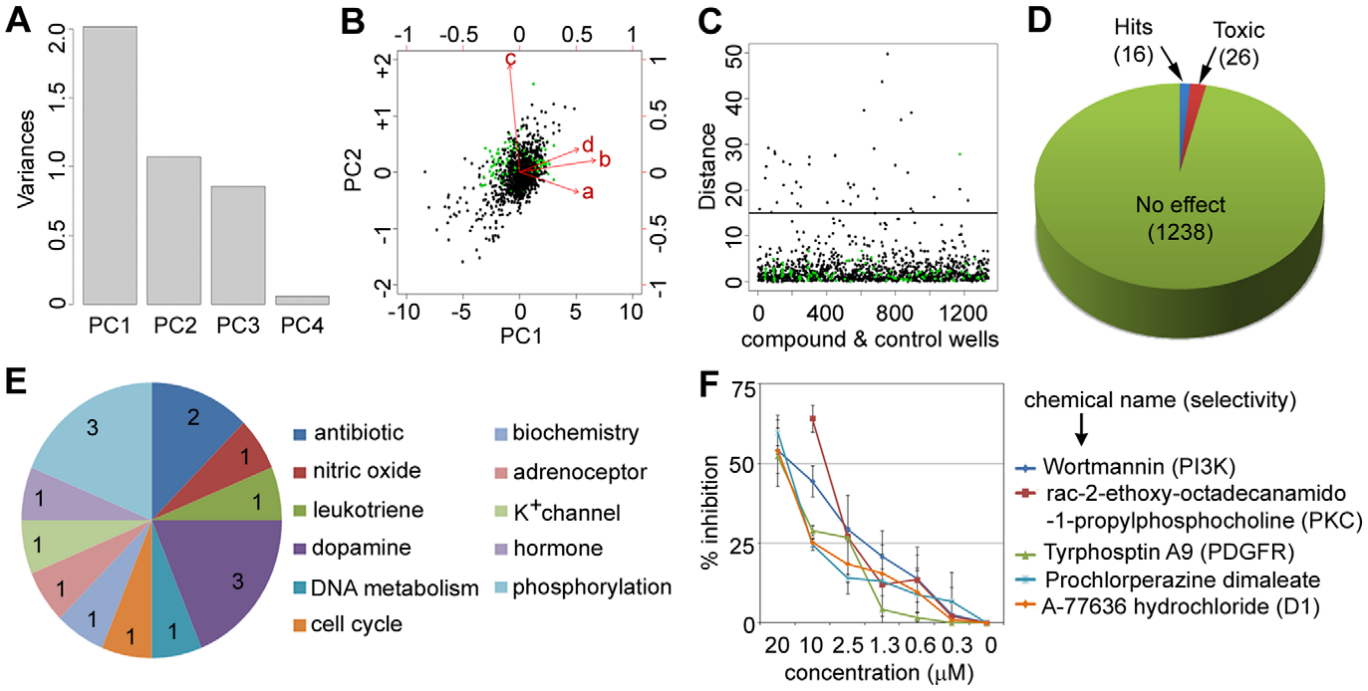
Phagocytosis screening assay results. (**A**) Variance accounted for by each of the principal components (PCs). (**B**) A biplot showing PC1 and PC2 for compounds (black) and DMSO controls (green). Input variables are represented by red vectors: a = number of cells Z-score, b = number of spots Z-score, c = spots per cell Z-score, and d = background-corrected spot signal per cell area Z-score. (**C**) Mahalanobis distance in PC1–PC2 space from each well to the medoid DMSO treated and infected control well. Compounds with Mahalanobis distance >15 and cell number Z score ≥ −3 were considered as hits. (**D**) A pie chart showing the number of hits (16) and cytotoxic compounds (26) from a total of 1280 compounds screened. (**E**) A pie chart showing the distribution of the identified hits based on their target classes. (**F**) Dose response curves of select inhibitors from the phagocytosis assay. The percent inhibition is the average ± standard deviation from duplicate wells of three independent experiments.

**Figure 3:**
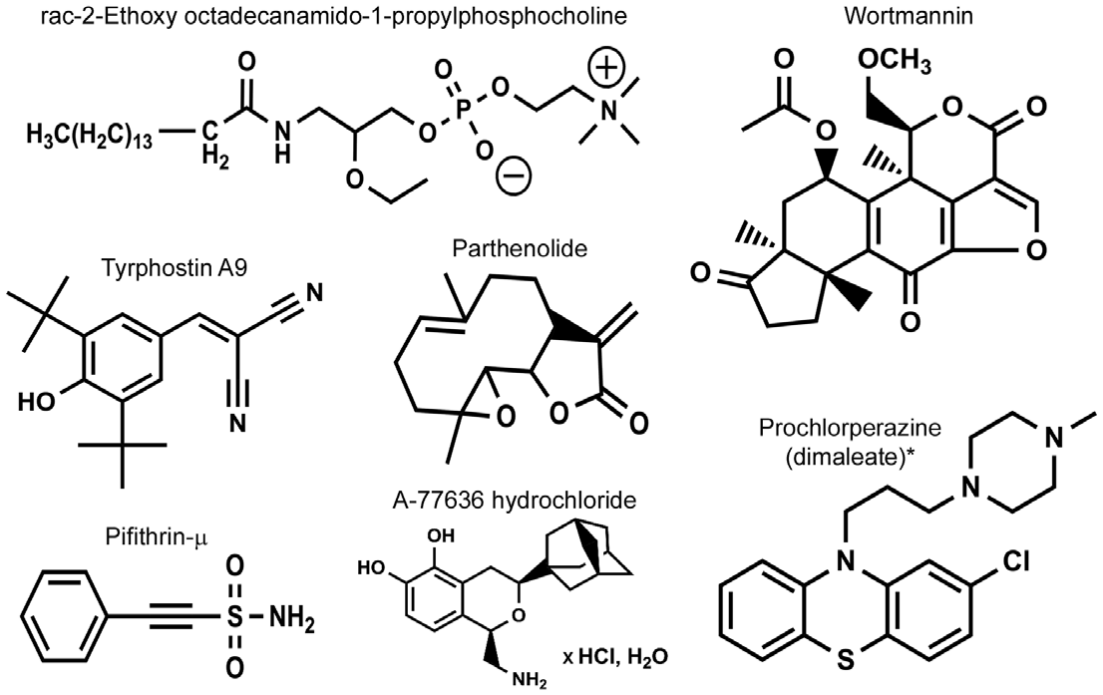
Chemical Structures. Structures of select ‘hit compounds’ identified during focused library screening. *For clarity, the structure of Prochlorperazine is shown in the organic form (versus the salts indicated in the ‘Compound Name’).

In the phagocytosis assay, the four output features described earlier were normalized across each plate and then subjected to principal components analysis. The amount of variance accounted for by the top four components is shown in **Figure 2A** **,** and the biplot in **Figure 2B** shows the variable rotations and first two principal components for infected wells. The distribution is approximately bivariate normal around zero, with control (DMSO-treated and infected) wells overlapping the main distribution. A Mahalanobis distance based thresholding method was then applied to the multivariate data set [21]. Mahalanobis distance from the medoid (multivariate median) DMSO-control and infected point is represented in **Figure 2C**, where only a single control point appears above the chosen cutoff distance of 15. A total of 16 bioactive small molecules that showed a Mahalanobis distance >15 and cell-number Z score ≥ -3 were considered hits (**Figure 2D** **and** **Table 1**). The identified bioactive compounds were associated with diverse target classes (**Figure 2E**), with half of the hits being distributed among three biological target classes: antibiotics, dopamine, and phosphorylation. The two known antibiotics identified in the phagocytosis screen also exhibited antibacterial activity in *in vitro* growth inhibition assays (data not shown). Retesting of the hit compounds from the phosphorylation and dopamine target class showed a dose-dependent inhibition of bacterial internalization for five of the six compounds (**Figure 2F**). The dopamine inhibitor thiothixene hydrochloride was a false positive as it did not show any significant inhibition in the dose-response studies. The chemical structures of the hit compounds are shown in **Figure 3**.

**Table 1:** Hits identified from the LOPAC^1280^ library screening in the phagocytosis assay and their corresponding target class and target selectivity.

Compound Name	Target Class	Target selectivity
Diphenyleneiodonium chloride	Nitric oxide	eNOS
Dequalinium chloride hydrate	K+ channel	_
rac-2-Ethoxy octadecanamido-1-propylphosphocholine	Phosphorylation	PKC
Wortmannin	Phosphorylation	PI3K
Tyrphostin A9	Phosphorylation	PDGFR
5-fluoro-5′-deoxyuridine	DNA metabolism	_
Doxycycline hydrochloride	Antibiotic	Protein synthesis
Ofloxacin	Antibiotic	DNA synthesis
Prazosin hydrochloride	Adrenoceptor	alpha1
5-Fluorouracil	Cell cycle	Thymidylate synthetase
Bestatin hydrochloride	Biochemistry	Aminopeptidase
*Thiothixene hydrochloride	Dopamine	D1/D2
A-77636 hydrochloride	Dopamine	D1
Prochlorperazine dimaleate	Dopamine	_
AC-93253 iodide	Hormone	‘RAR(a)
Ebselen	Leukotriene	–

* False positive.

### Quantitative Imaging of Y. Pestis-induced Host Nuclear Factor-κB (NF-κB) Activation and Translocation

**Figure 4:**
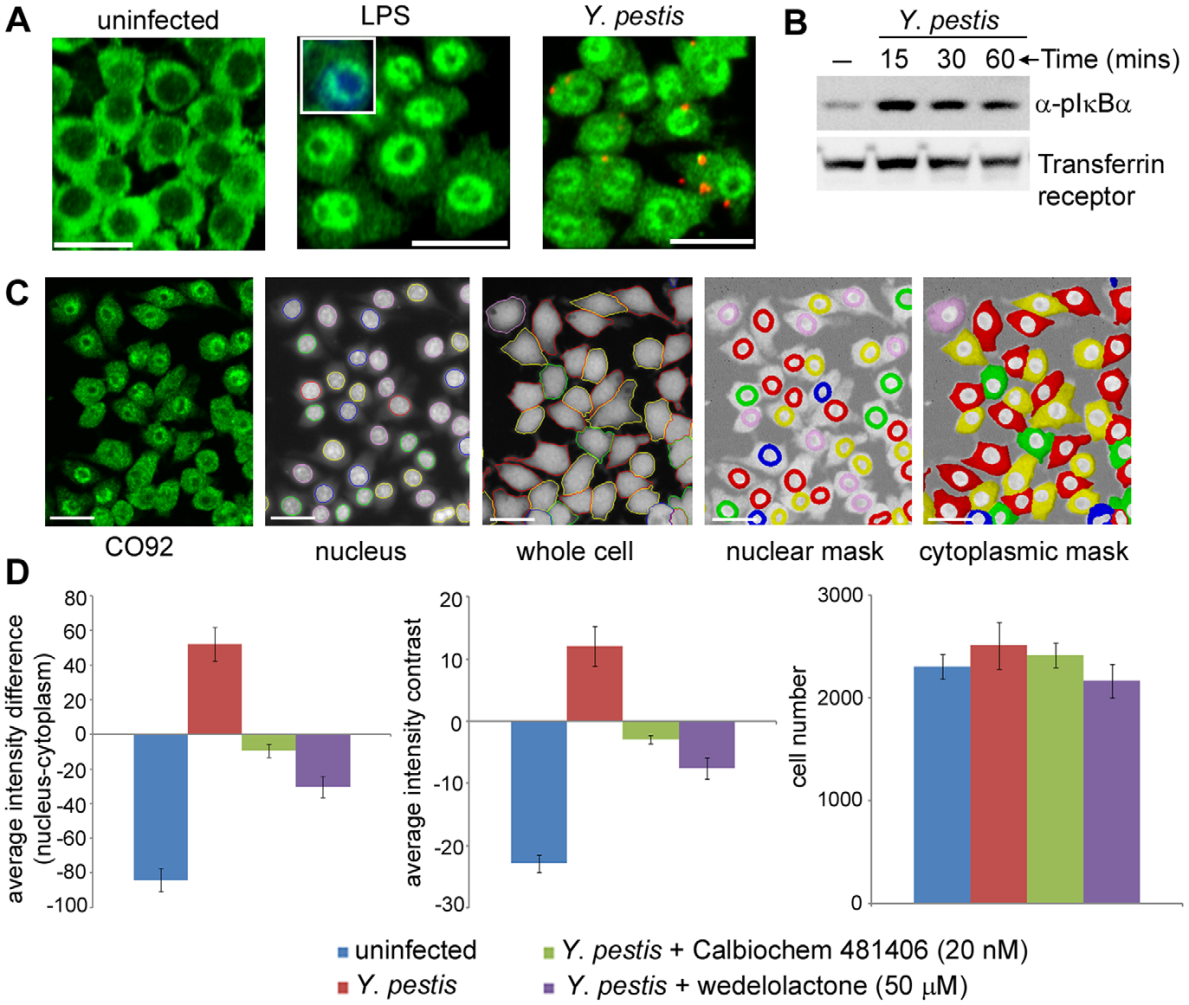
Quantitative imaging of NF-κB activation and translocation in macrophages. (**A**) NF-κB activation and translocation. RAW264.7 macrophages were either left untreated (left panel) or treated with LPS (middle panel) or infected with 10∶1 MOI of *Y. pestis* CO92 strain (right panel). Green – NF-κB stained with αNF-κB antibody; Red – *Y*. *pestis* stained with α FI antibody. The inset picture (middle panel) shows staining of the nucleus with the Hoechst nuclear dye and the nuclear localization of NF-κB stained in green with αNF-κB antibody. Scale bar = 20 µm. (**B**) Western blot analysis. Cell lysates from *Y*. *pestis* infected macrophages were subjected to Western blotting and probed with α pIκBα (Ser 32) antibody or transferrin receptor antibody as loading control. (**C**) Image segmentation of NF-κB activation and translocation. Images acquired in the UV channel (Hoechst stain) were segmented to mark the nuclear boundaries (nucleus), and images acquired in a 640 nm channel (CellMask Deep Red dye) were segmented to define the cell boundaries (whole cell). Masks were created for individual objects using the boundaries defined by the nucleus (nuclear mask) and cytoplasm (cytoplasmic mask) channels. (**D**) Quantitation of NF-κB nuclear translocation. Graphical representations of two image output features, which depict the distribution of NF-κB between the cytoplasm and the nucleus following different stimuli. The average intensity difference (nuclear intensity – cytoplasmic intensity) and average intensity contrast ((nuclear intensity – cytoplasmic intensity)/nuclear+cytoplasmic intensity*100)) results have been shown. The graphical representation of the average cell number, from all the imaged fields, is shown in the right panel. The data is the average of four replicates ± standard deviation, and is representative of three independent experiments.

Automated HCI assays can also be applied to quantitate host responses to *Y. pestis* infection. For example, activation of the NF-κB pathway constitutes an important part of the host defense against multiple pathogens [22]. Upon exposure to *Yersinia*, macrophages activate the NF-κB pathway. *Yersinia* interferes with this pathway by secreting the effector protein YopJ, and thereby inducing the apoptosis of infected macrophages [23], [24]. However, this role of *Y. pestis* is uncertain as *Y. pestis* YopJ mutants are reported to have no defect in virulence *in vivo* [25]. Activation of NF-κB requires the phosphorylation of IκBα, which then targets IκBα for ubiquitination and proteasome-mediated degradation, thereby releasing NF-κB to translocate to the nucleus where it activates several genes involved in the inflammatory response [26]. To quantitate the activation and translocation of NF-κB, RAW264.7 macrophages were either treated with a known inducer, *Escherichia coli* K12 lipopolysaccharide (LPS) or infected with 10∶1 MOI of *Y*. *pestis* CO92. Endogenous NF-κB was then visualized by immunofluorescence using αNF-κB antibody. In non-stimulated cells, NF-κB was sequestered in the cytoplasm (**Figure 4A, left panel**). Upon stimulation for 30 min with either LPS (**Figure 4A** **, middle panel**) or infection with *Y. pestis* (**Figure 4A, right panel**), NF-κB was translocated to the nucleus. However, at 30 mins post-infection, very few bacteria (Alexa 568 staining) that were cell-associated or internalized were visible. Western blot analysis of *Y*. *pestis* infected macrophages showed increased phosphorylation of inhibitor protein IκBα versus uninfected controls (**Figure 4B**), thus confirming our observation from the HCI studies, that during the early stages of infection, *Y. pestis* induces phosphorylation of IκBα, which leads to NF-κB activation and translocation.

To quantitate NF-κB nuclear translocation, images were computationally analyzed using an automated image-analysis translocation script. The nuclei and cell boundaries were segmented based on the Hoechst 33342 DNA stain and the CellMask Deep Red stain respectively (**Figure 4C**) and NF-κB was visualized using αNF-κB antibody. During the image analysis, several features were captured (**Table S2**). The nuclear NF-κB translocation event was measured by determining: 1) the average signal intensity difference between the nucleus and cytoplasm regions; and 2) the average intensity contrast (see Materials and Methods for details). The chemical inducers LPS and Pam3CSK4 (a TLR2 agonist) and attenuated (**Figure S2A**) or virulent *Y. pestis* CO92 (**Figure 4D**) were capable of promoting nuclear NF-κB translocation 30 min post-treatment, as measured by a quantitative increase in the signal intensity difference and signal intensity contrast between the nucleus and the cytoplasm. The untreated cells showed greater signal intensity in the cytoplasm than the nucleus, resulting in a negative value. RAW264.7 macrophages infected with varying MOI (10∶1, 30∶1 and 50∶1) for 30 min showed similar activation and nuclear translocation of NF-κB (**Figure S2B)**. Longer incubation with LPS or *Y. pestis* CO92 resulted in changes in the dynamics of NF-κB redistribution. Specifically, an oscillation pattern was observed in the temporal response of NF-κB activation (**Figure S3**), a feature similar to that observed in reporter cell lines following stimulation with TNF-α, LPS and IL-1β or infection with *Helicobacter pylori* [27].

To test the utility of the high-content NF-κB translocation assay for chemical screens, RAW264.7 macrophages were pretreated with two known inhibitors of NF-κB transcriptional activity and then infected with *Y. pestis* CO92. As shown in **Figure 4D**, calbiochem 481406 (at low nM concentrations) and wedelolactone (at high µM concentrations) inhibited pathogen induced NF-κB translocation. Furthermore, the robustness of the assay was evaluated by calculating the Z′ value, which yielded values between 0.5–0.6 for the different inducers.

Overall, the results indicate that the distribution of endogenously expressed NF-κB, which is indicative of its activity, can be quantitatively analyzed in a heterogeneous cell population using an automated HCI assay, which can then be applied to identify small molecules that alter this signaling process.

### Screening of Pharmacologically Active Compounds in the NF-κB Nuclear Translocation Assay

To examine the effects of small molecules on *Y. pestis* induced NF-κB nuclear translocation, the Sigma-Aldrich LOPAC^1280^ library was screened at a single concentration (20 µM) in the optimized NF-κB nuclear translocation assay. For each plate, values were normalized to the median of all infected wells in the plate. Principal components analysis was then applied to combine the multiple output features extracted during image analysis related to *Y*. *pestis* induced NF-κB activation and translocation.

**Figure 5:**
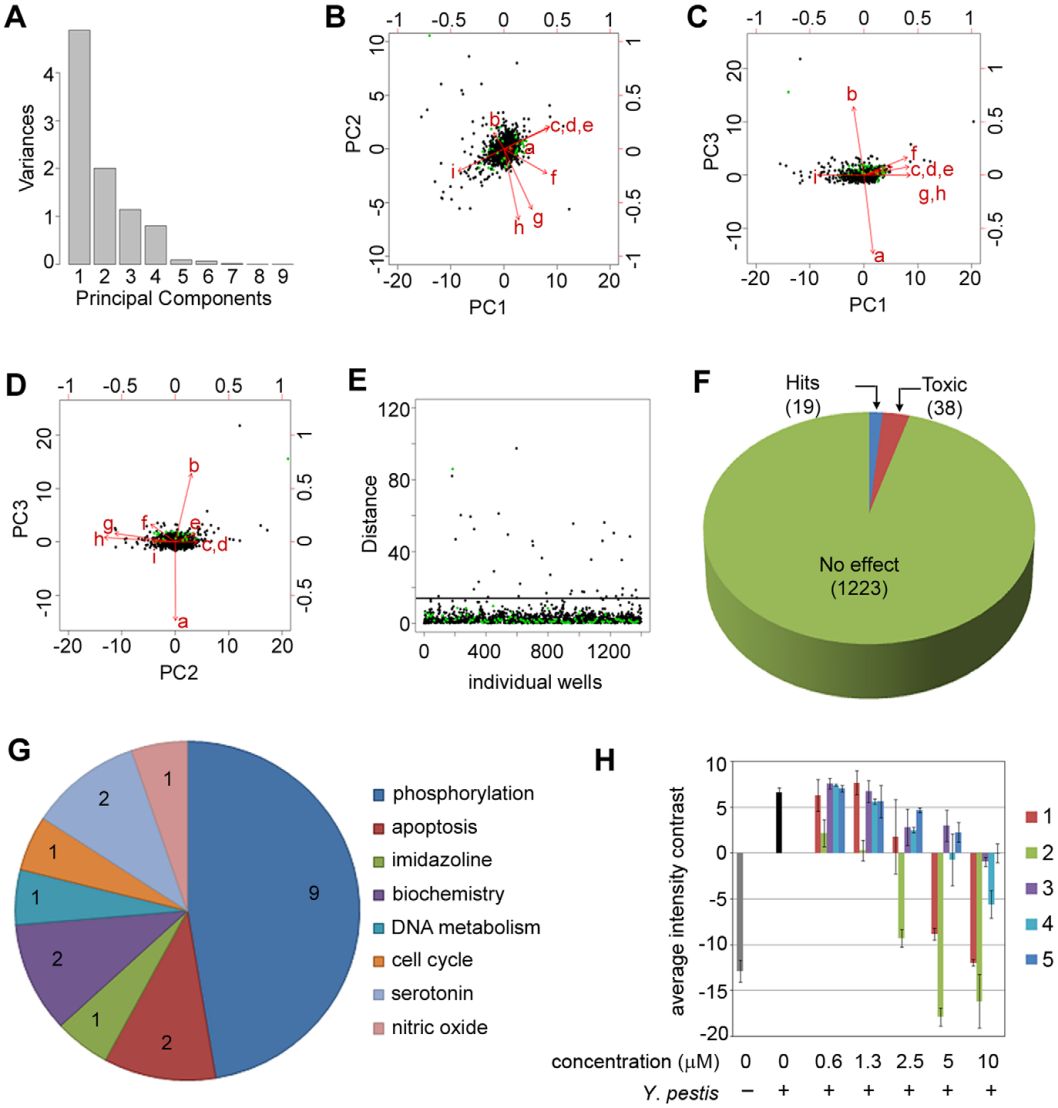
NF-κB screening assay results. (**A**) Variance accounted for by each of the principal components (PCs). (**B–D**) Scatter plots showing PC1 and PC2 (**B**), PC1 and PC3 (**C**), and PC2 and PC3 (**D**) for compounds tested (black) and DMSO controls (green). Input variables are represented by red vectors: a = number of cells Z-score, b = nucleus size Z-score, c = intensity contrast Z-score, d = normalized nucleus intensity Z-score, e = intensity ratio Z-score, f = intensity difference Z-score, g = nucleus intensity Z-score, h = cytoplasm intensity Z-score, and i = normalized cytoplasm intensity Z-score. (**E**) Mahalanobis distance in PC1-3 space from each well to the medoid DMSO control well. Compounds with Mahalanobis distance >15 and cell number Z score ≥ −3 were considered hits. (**F**) A pie chart showing the number of hits (19) and cytotoxic compounds (38) from a total of 1280 compounds screened. (**G**) A pie chart showing the distribution of the identified hits based on their target classes. (**H**) Dose response curves of select inhibitors of the NF-κB translocation assay. 1 - rac-2-Ethoxy-3 octadecanamido-1-propylphosphocholine; 2– parthenolide; 3– wortmannin; 4– pifithrin-mu; 5– tyrphostin A9. The data is the average of two replicates ± standard deviation, and is representative of three independent experiments.

During analysis of the screening data from the NF-κB activation assay, nine output features (**Table S2)** were normalized across each plate and used in principal components analysis. **Figure 5A** shows the level of variance accounted for by each of the nine components, and the biplots in **Figure 5B–D** show the variable rotations and first three principal components for infected wells. The DMSO-treated and infected control wells were centered at zero. The multivariate Mahalanobis distance measure was determined from the medoid for DMSO-treated and infected wells and is shown in **Figure 5E**. Only one control well displayed a distance greater than the indicated cutoff of 15. Bioactive compounds that showed a Mahalanobis distance >15 and a cell number Z score ≥ −3 were considered hits. A total of 19 hits were identified (**Figure 5F** **and** **Table 2**) with the majority again being associated with the phosphorylation target class (**Figure 5G**). Out of the 19 hits identified, 17 compounds inhibited *Y. pestis* induced NF-κB translocation, whereas two compounds – ellipticin, a Cytochrome P450 (CYP1A1) and DNA topoisomerase II inhibitor, and PD 169316, a selective p38 kinase inhibitor, increased pathogen induced NF-κB activation compared to the infected control. However, upon further investigation, both activators were determined to be false positives, as they caused interference in the channel used to detect NF-κB signal (488 nm). The compounds that showed the strongest inhibition from representative classes namely- phosphorylation (rac-2-Ethoxy-3-octadecanamido-1-propylphosphocholine and wortmannin), serotonin (parthenolide) and apoptosis (pifithrin-mu) were further confirmed in a dose response study (**Figure 5H**). Tyrphostin A9, which inhibited *Y*. *pestis* internalization (**Figure 2**), but fell short of the Mahalanobis cut-off in the pathogen-induced NF-κB translocation assay, was nevertheless retested in the dose response study.

**Table 2:** Hits identified from the LOPAC^1280^ library screening in the *Y. pestis* induced NF-κB assay.

Compound Name	Target Class	Target selectivity
Ellipticine*	Cell cycle	CYP1A1/TopoII
PD 169316*	Phosphorylation	p38 MAP kinase
rac-2-Ethoxy-octadecanamido-1-propylphosphocholine	Phosphorylation	PKC
SU 6656	Phosphorylation	Src family
Wortmannin	Phosphorylation	PI3K
Bay 11-7082	Phosphorylation	IKB-alpha
ZM 39923 hydrochloride	Phosphorylation	JNK-3
NSC 95397	Phosphorylation	Cdc25
‘BIO	Phosphorylation	GSK-3alpha/beta
Tyrphostin AG 698	Phosphorylation	EGFR
SB 204070 hydrochloride	Serotonin	5-HT4
Parthenolide**	Serotonin/Phosphorylation	IκB kinase [29]
Pifithrin-mu	Apoptosis	P53 binding
Imiquimod	Apoptosis	Caspase 3
Tetraethylthiuram disulfide	Biochemistry	Alcohol Dehydrogenase
Idazoxan hydrochloride	Imidazoline	I1/I2
Z-L-Phe chloromethyl ketone	Biochemistry	Chymotrypsin A-gamma
Mitoxantrone	DNA metabolism	
S-Nitroso-N-acetylpenicillamine	Nitric oxide	

* The two compounds potentiated *Y. pestis* induced NF-κB activation, which was due to their interference in the signal channel.

** Compound parthenolide is classified as belonging to the serotonin class in the LOPAC library. Published studies by Saadane *et al* [29] reveal that parthenolide is an IκB kinase inhibitor and hence included in the phosphorylation target class.

In summary, our screening of the LOPAC library in the phagocytosis and NF-κB assays identified two kinase inhibitors, rac-2-Ethoxy-3-octadecanamido-1-propylphosphocholine and wortmannin, which strongly inhibit *Y. pestis* internalization and pathogen-induced NF-κB activation during the early stages of infection. The compounds parthenolide (from the serotonin class) and pifithrin-mu (an inhibitor of p53 binding to mitochondria [28] and grouped in the apoptosis class), both inhibited *Y. pestis*-induced NF-κB activation but did not inhibit bacterial internalization. Previously, parthenolide was shown to inhibit IκB kinase and the subsequent activation of NF-κB [29].

### Inhibition of NF-κB Activation by Select Hit Compounds is Dependent on the Applied Inducer

**Figure 6:**
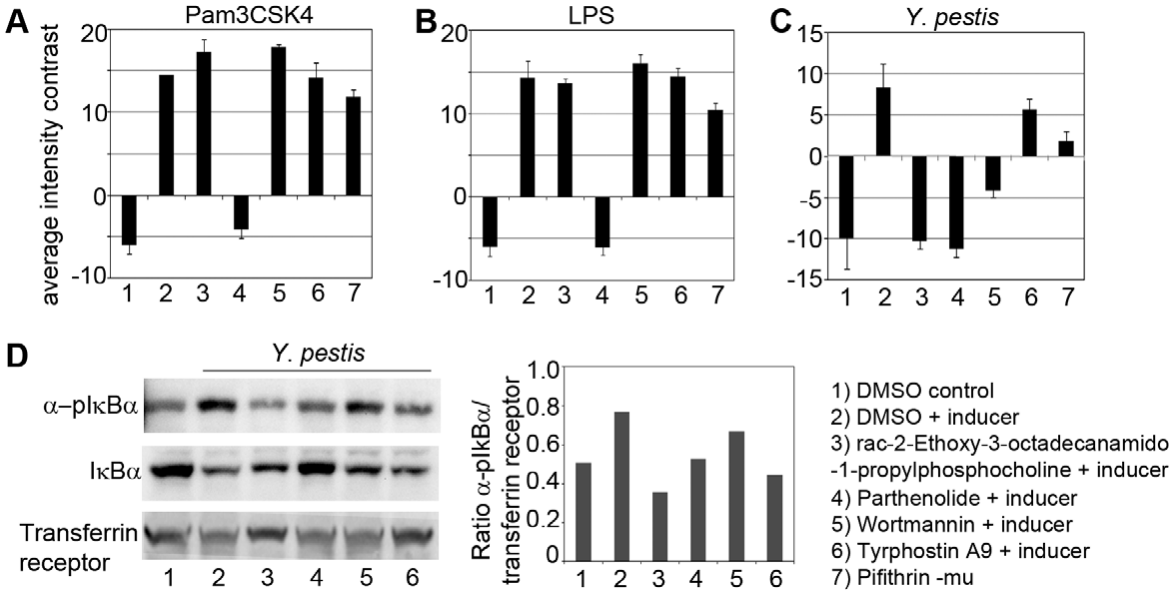
Inhibition of early NF-κB activation by select compounds is dependent on the applied inducer. Raw264.7 macrophages pretreated for 2 hr with: 1 & 2) DMSO control; 3) rac-2-Ethoxy-3 octadecanamido-1-propylphosphocholine; 4) parthenolide; 5) wortmannin; 6) tyrphostin A9; 7) pifithrin-mu, and then treated for 30 min with inducers (lanes 2–7) (**A**) Pam3CSK4 (1 µg/ml); (**B**) LPS (1 µg/ml); and (**C**) *Y*. *pestis* CO92 (10∶1 MOI). The data is the average of six replicates ± standard deviation, and is representative of three independent experiments. (**D**) Western blot analysis of cell lysates obtained from macrophages pretreated for 2 hr with: 1 & 2) DMSO control; 3) rac-2-Ethoxy-3 octadecanamido-1-propylphosphocholine; 4) parthenolide; 5) wortmannin; 6) tyrphostin A9 and then infected for 30 min with *Y. pestis* (lanes 2–6). In the left panel, the blots were probed with α pIκBα (Ser 32) antibody to measure IκBα phosphorylation and IκBα antibody to measure IκBα degradation. Transferrin receptor antibody was used as loading control. The right panel is a bar graph of the densitometric scan of the pIκBα and transferrin receptor bands and is depicted as a ratio of the pixel intensities for the two bands.

To determine if the inhibition of NF-κB activation by the identified, hit compounds was independent of the applied stimulus (*i.e*. inducer), RAW264.7 macrophages were pretreated with the select bioactive compounds and then stimulated for 30 min with a TLR2 agonist (Pam3CSK4) (**Figure 6A**), TLR4 agonist (*E. coli* K12 LPS) (**Figure 6B**) or infected with 10∶1 MOI of *Y. pestis* CO92 (**Figure 6C**). NF-κB translocation induced by LPS or Pam3CSK4 was not inhibited by rac-2-Ethoxy-3 octadecanamido-1-propylphosphocholine, wortmannin or pifithrin-mu but was inhibited by parthenolide. On the contrary all four compounds inhibited NF-κB translocation induced by *Y. pestis*. The compound tyrphostin A9 was a weak inhibitor of *Y. pestis* induced NF-κB translocation that also did not inhibit LPS or Pam3CSK4 mediated NF-κB translocation. The inhibition of *Y. pestis* CO92-induced NF-κB activation by the select compounds was further confirmed by immunoblot analysis (**Figure 6D**, left panel) and subsequent densitometry scans of pIκBα and transferrin receptor as loading control (**Figure 6D, right panel**). As shown in **Figure 6D**, the compounds slightly reduced *Y. pestis* mediated IκBα phosphorylation and degradation. These results suggest that activation of NF-κB *by Y. pestis* CO92 occurs during phagocytosis at the early stages of infection.

### Regulation of Y. Pestis Infection by Select Bioactive Compounds

**Figure 7:**
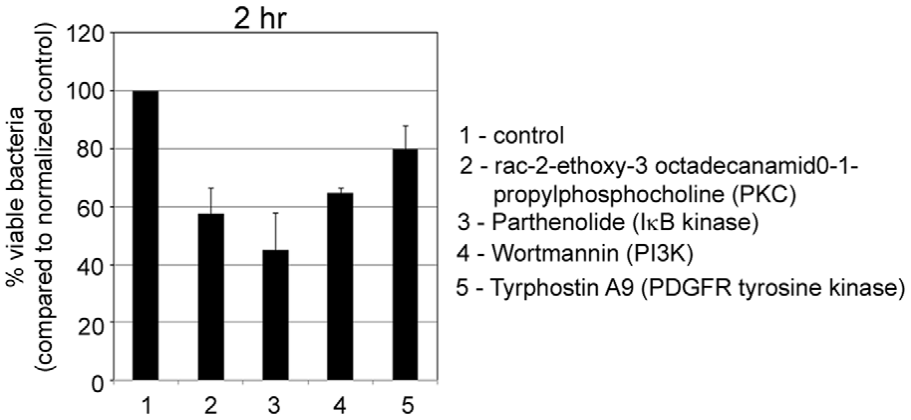
Regulation of *Y*. *pestis* infection by select hit compounds. A bar graph showing the percentage of viable bacteria in RAW264.7 macrophages treated with DMSO control (and normalized to 100% viable bacteria) or 10 µM of the compounds (except Wortmannin which was applied at 20 µM). Bacterial viability was determined at 2 hr following infection. The percentage of viable bacteria is the average ± standard deviation from two replicates of two independent experiments.

To determine the effect of the select compounds on bacterial viability, RAW264.7 macrophages were pre-treated with select compounds and infected with *Y*. *pestis* CO92 at an MOI of 30∶1. At 2 hr post-infection, the macrophages were lysed and plated on blood agar plates. Enumeration of the colony counts showed a statistically significant reduction in number of viable bacteria following pre-treatment of macrophages with compounds that selectively target PI3K, and PKC (p<0.05) but not PDGFR tyrosine kinase (**Figure 7**). This result demonstrates that the tested compounds may either be affecting bacterial infection or enhancing macrophage bactericidal activity, both of which may result in reduced number of viable bacteria. Surprisingly, the compound parthenolide (previously reported to target IκB kinase [29]), which inhibited pathogen induced NF-κB activation, but did not significantly inhibit internalization of *Y*. *pestis* CO92 by macrophages, was also found to cause a significant reduction (p<0.05) in the number of viable bacteria (**Figure 7**). The identified compounds do not exhibit antibacterial activity when tested directly on the bacteria in the *in vitro* growth inhibition assays (**Figure S4**) and bacterial viability assays (**Figure S5**) and also do not exhibit cytotoxic effects on macrophages as measured using cell-based viability assays (**Figure S6**).

## Discussion

Although the existence of an intracellular phase in the life cycle of *Y. pestis* remains a matter of debate, several studies [30]–[35] along with our *in vitro* EM and HCI results (Figure1) indicate the presence of intracellular bacteria within host macrophages during the early stages of infection. *Y. pestis* can also replicate efficiently in the extracellular environment, and hence, in all of the experiments carried out in this study, careful attention was paid to remove cell-associated and non-internalized bacteria by using stringent washing conditions. Application of the stringent washing conditions and studies using dual labeled bacteria (*i.e* bacterial staining without the permeabilization of macrophages) revealed that while a vast majority of the detected bacteria are internalized, about 15% of bacteria still remain cell-associated and external.

In this study we developed two high-throughput image-based cellular assays to quantitate bacterial phagocytosis and activation of the host NF-κB signaling pathway in response to *Y. pestis* exposure. Pharmacologically active small molecules were then used as probes to elucidate the cellular pathways that regulate the host-pathogen interactions. Screening of 1280 bioactive small molecules in the two HCI assays resulted in the identification of 15 small molecules (and one false positive) that reduced *Y. pestis* cell-association and internalization, and.17 compounds (and two false positives) that inhibited pathogen-induced, host NF-κB activation and translocation, during the early stages of *Y. pestis* infection. Interestingly, a majority of the hits identified in both the HCI assays were kinase inhibitors. The possible concern that curing of the pCD1 virulence plasmid during macrophage infection may increase phagocytosis and NF-κB activation and thus bias our screen to weaker inhibition. However, the PCR detection of the pCD1 plasmid in infected RAW264.7 macrophage lysates suggests otherwise.

In the phagocytosis HCI assay, the three kinase inhibitors, rac-2-Ethoxy-3 octadecanamido-1-propylphosphocholine, wortmannin and tyrphostin A9 (which that selectively target PKC, PI3K and PDGFR tyrosine kinases, respectively (Table 1) showed dose-dependent inhibition of *Y*. *pestis* cell-association and internalization and subsequent reduction in the number of viable bacteria, in the colony-forming assay. Prior studies using non-phagocytic HeLa cells have shown that protein kinase inhibitors wortmannin, genistein, and staurosporine decreased the invasion of *E.coli* expressing *Y*. *pestis* plasminogen activator [18]. Internalization of *E.coli* expressing the *Y*. *enterocolitica* invasion gene *inv*, by HeLa cells can also be inhibited by genistein, staurosporine and tyrphostin AG34 [36]. While *Y*. pestis lacks the adhesion proteins invasin and YadA, it does express several other adhesion proteins. However, specific characterization of their adhesion properties and subsequent contribution to enteric infection is still poorly understood [37]. The interaction of *Y*. *pestis* adhesion proteins with eukaryotic host cell integrin receptors enables the bacteria to adhere to the cells and modulate intracellular signals that promote cytoskeletal rearrangement which is required for bacterial internalization [38]. In this study we demonstrated that macrophages pretreated with cytochalasin D reduced *Y. pestis* CO92 binding and uptake, as indicated by our HCI phagocytosis assay and by a reduction in the number of viable bacteria within infected macrophages as determined by the intracellular replication assay (data not shown).

Two hits from the dopamine target class (Table 1) also exhibited dose-dependent inhibition of *Y. pestis* phagocytosis in macrophages. Dopamine receptor antagonist prochlorperazine dimaleate is an anti-psychotic drug that has been shown to exhibit modest broad-spectrum antibacterial activity [39] and potentiates the antimicrobial properties of a wide-spectrum of antibiotics against several Gram-positive and Gram-negative bacteria [40], [41], possibly by interfering with bacterial encoded efflux pump(s) [42]. The compound A-77636 hydrochloride which was also identified as a hit in our assay is an orally active dopamine receptor agonist that is structurally very different from prochlorperazine dimaleate. To date, there is no report of this compound exhibiting antibacterial activity or modulating bacterial internalization. While further characterization of hit compounds from the dopamine class need to be pursued, none of the five inhibitors identified in the phagocytosis HCI assay demonstrated *in vitro* antibacterial activity when tested at 20 µM concentrations.

The *Y. pestis* virulence factor YopJ is a potent inhibitor of the NF-κB signaling pathway [24], [43], [44]. Characterization of effector protein functions have generally been performed by over expressing the virulence plasmid in non-phagocytic or phagocytic macrophages and measuring the effects on the signaling pathways or downstream cytokine secretions [43], [45]. In our studies, RAW264.7 macrophages infected with the virulent *Y. pestis* CO92 and fixed at different time points showed an oscillating temporal response to NF-κB activation and translocation (Figure S3). A similar response pattern has previously been reported using cell lines expressing the p65-GFP reporter and stimulated with various stimuli such as TNF-α, LPS and IL-1β [27]. Additionally, in the same study a dampened oscillation of p65 translocation was also observed in the reporter cell lines following infection with the bacterium *Helicobacter pylori* [27]. The combination of live-cell imaging and computational modeling at the single cell level has revealed that cells with identical genotypes generate a qualitatively diverse NF-κB activation response to the same stimulus [46]. These diverse responses may help support the wide range of roles that NF-κB plays in regulating processes such as cell proliferation, apoptosis, innate immunity, and inflammatory responses. Therefore, single-cell analysis of *Y*. *pestis* induced NF-κB oscillation patterns will further aid the fundamental effort towards understanding how individual cells respond to not only infection by *Y. pestis* but bacterial infection in general.

Screening of the LOPAC^1280^ bioactive compound library using our NF-κB HCI assay identified 17 small molecules that inhibited *Y. pestis* induced early NF-κB activation. Two kinase inhibitors, rac-2-Ethoxy-3 octadecanamido-1-propylphosphocholine (PKC inhibitor) and wortmannin (PI3K inhibitor) were identified as hits in both the phagocytosis and NF-κB HCI assay. The compounds parthenolide, a known inhibitor of IκB kinase [29] and pifithrin-mu, a known inhibitor of p53 binding and apoptosis [28] both inhibited *Y. pestis* induced early NF-κB activation. However, the mechanism through which they regulate bacterial entry and survival requires further characterization. The compound parthenolide inhibited NF-κB activation and translocation in response to stimulation with Pam3CSK4 (TLR2 agonist), *E. coli* LPS (TLR4 agonist) or *Y. pestis* infection, while the PI3K and PKC inhibitors were selective only for *Y. pestis*, thus suggesting that phagocytosis is the major stimulus for NF-κB activation in response to *Y. pestis* infection.

In summary, we have developed multiple HCI assays to quantitate phagocytosis and host responses during infection of macrophages with the virulent *Y. pestis*. Chemical genetic screening was then applied to these assays to probe host signaling pathways that regulate *Y. pestis* infection. Such host-directed integrated screening strategies can be applied to other bacterial pathogens to elucidate mechanisms of bacterial pathogenesis and host cell defense(s). Adopting these technologies for dynamic live imaging will provide enhanced capabilities that will facilitate the more rapid dissection of the sequence of events that unfold as bacteria interface with, and infect host cells, thereby aiding in the identification of much needed antibacterial agents with novel mechanisms of action.

## Materials and Methods

### Bacterial Pathogens and Growth Conditions

Avirulent *Y. pestis* CO92 (Pgm^−^, pPst^−^), that is pigmentation (*pgm*)-deficient and cured of the plasminogen-activator-encoding pPst plasmid (a gift from Drs. Susan Welkos and Christopher Cote, USAMRIID, [47], [48] ) and virulent *Y. pestis* CO92 were used in these studies. For all macrophage infection studies, *Y. pestis* strains from frozen stocks were streaked onto sheep blood agar (SBA) plates and incubated at 35°C for 24 hr, unless otherwise noted. The next day, bacterial colonies from the plates were resuspended in mammalian tissue culture medium (DMEM medium containing 10% FBS, 1% non-essential amino acids and 1% L-glutamine and referred to as complete DMEM medium), absorbance at 600 nM (OD_600_) was measured and bacterial concentrations were determined based on a *Y. pestis* standard growth curve generated from colony forming unit (CFU) assays. Bacteria were then further diluted in complete DMEM medium to appropriate concentration based on the individual assay requirements. *Y. pestis* was grown at 35°C to get optimal staining of the bacteria with the αFI monoclonal antibody (F1-04-A-G1) [17] during infection of macrophages. The presence of the *Y. pestis* plasmids pPCP1 (primer and probes targeting *pim* and *pla*) and pFra (gene encoding F1) were confirmed by real time PCR [49] (**Table S3**). The presence of pCD1 virulence plasmid was confirmed by PCR analysis followed by agarose gel electrophoresis of the amplified product (**Figure S7**). Nucleic acid was extracted from bacteria grown on SBA plates incubated at either 28°C or 35°C for 24 hr as well as from *Y. pestis* infected macrophages 2 hr post infection.

For dual labeling studies, *Y. pestis* CO92 derivative expressing GFP in trans was constructed. Briefly, *Y. pestis* (Pgm− and Pla−) was electroporated with plasmid pFVP 25.1 (G. Mallo Caenorhabditis Genetics Center) using the methods described by Conchas and Carniel [50]. *Y. pestis* was cultured in 5 ml of Luria-Bertani (LB) broth (Sigma, St. Louis, MO) overnight at 28°C with aeration. The overnight culture was added to 25 ml of LB broth and incubated until the OD_600_ reached ∼0.5. The culture was harvested at 6,000 rpm for 8 min, supernatant decanted, and the cell pellet was washed with 10 ml of sterile water. The cell re-suspension was centrifuged as described above and washed with 10 ml of transformation buffer (272 mM sucrose in 15% glycerol), centrifuged, supernatant decanted, and re-suspended in 1 ml of transformation buffer. Electroporation was performed by combining 40 µl of the competent cells and 0.5 µg of plasmid DNA followed by a 10 min incubation on ice. Cells were transferred into a chilled 0.1 cm cuvette and electroporated by delivering a single pulse of 2.5 kV, 25 µF, and 200 Ω using a GenePulser Xcell electroporator (BioRad, Hercules, CA). Cells were immediately added to 1 ml of SOC medium (Sigma) and recovered for 1 hr at 28°C with aeration (250 rpm). Aliquots (50 µl) were plated onto LB plates containing 100 µg/ml ampicillin (Sigma) and incubated for 48 hr at 28°C. For macrophages infection studies, *Y. pestis* CO92 derivative expressing GFP was first grown on blood agar plate and incubated overnight at 35°C. Bacterial colonies were resuspended in complete DMEM medium, OD_600_ measured, and cell suspensions were diluted to obtain the targeted MOI.

### Cell Culture

For all studies, RAW264.7 murine macrophage-like cell line (ATCC TIB-71) was used and referred throughout in the text as RAW264.7 macrophages. The cells were grown at 37°C and 5% CO_2_ in DMEM medium containing 10% FBS, 1% non-essential amino acids and 1% L-glutamine and referred to as complete DMEM medium.

### Small Molecule Library

The Sigma-Aldrich LOPAC^1280^ library which contains pharmacologically active compounds was used for screening in the bacterial infection assays. The compounds were tested at 20 µM concentration in the primary screening assays.

### Transmission Electron Microscopy

RAW264.7 macrophages (ATCC TIB-71) were infected at a multiplicity of infection (MOI) of 30 *Y. pestis* CO92 bacteria to 1 macrophage (30∶1) for 2 hr or 8 hr at 37°C and 5% CO_2_. The cells were washed three times with phosphate buffered saline (PBS) to remove extracellular bacteria and fixed for a minimum of 24 hr in 0.1M PBS buffer containing 1% glutaraldehyde (Sigma, St Louis, MO) and 4% paraformaldehyde (Sigma). The cells were washed with PBS and then incubated for 1 hr in 1% osmium tetroxide (EMS, Hatfield, PA) prepared in PBS. Prior to further contrasting, cells were buffer washed, rinsed in 50% ethanol, contrasted in ethanolic uranyl acetate, and dehydrated in an ascending series of ethanol. Propylene oxide (PO; EMS) was utilized as a transition solvent and samples were infiltrated in 100% PO, a 50∶50 mix of PO and EMbed-812 epoxy resin (EMS), and pure EMbed-812. Samples in pure resin were polymerized by incubation at 60°C for not less than twenty-four hours. Embedded samples were sectioned at ∼70 nm on a Leica ultramicrotome, mounted on copper mesh grids, and contrasted with uranyl and lead salts (EMS). Sections were examined on a JEOL 1011 transmission electron microscope at 80 kV. Digital images were acquired using a Hamamatsu ORCA-HR digital camera controlled by AMT image acquisition software. Images were cropped and globally adjusted for contrast using Adobe Photoshop.

### Phagocytosis Assay

RAW264.7 macrophages were seeded at a density of 40,000 cells/well in 96-well imaging plates (Whatman 7716-2370 or BD 353219 imaging plates) and incubated at 37°C and 5% CO_2_. The next day, cells were pre-treated with 0.5% DMSO or 3 µM cytochalasin D (inhibitor control, Sigma C2618) or 20 µM of compounds (100 µl/well) from sigma LOPAC^1280^ library. After incubation for 2 hr at 37°C and 5% CO_2_, appropriate wells were infected with MOI of 30∶1 (or unless otherwise noted) with *Y. pestis* CO92. For assay development and optimization, avirulent *Y. pestis* (Pgm^−^, pPst^−^) was used. After incubation for 2 hr at 37°C and 5% CO_2_, the cells were washed 2 times with phosphate buffered saline (PBS), and uptake of the bacteria was inhibited by fixing the cells with 10% formalin for 30 min (if using the avirulent strain) or 48 hr (if using the virulent strain). After washing, the cells were permeabilized for 15 min with Dulbecco’s PBS containing 1% TX-100, washed and then blocked for 1 hr with blocking buffer (Cellomics 1860291). Bacteria were detected by incubating the infected cells for 1 hr with *Y. pestis* αF1, (2 µg/ml in blocking buffer), an IgG monoclonal antibody that is specific for the *Y. pestis* capsule antigen. After subsequent washing and incubation for 1 hr with the anti-mouse Dylight 488 secondary antibody (1∶500 dilution in blocking buffer), the cells were stained with Hoechst nuclear dye (Invitrogen H3570, 1 µg/ml in PBS) and CellMask Deep Red dye (Invitrogen H32721, 5 µg/ml in PBS) for host cell cytoplasmic staining.

For dose response studies, RAW264.7 macrophages were pretreated for 2 hr with 2-fold serial dilutions of select small molecules that induced a cellular phenotype in the primary screen. Cells were then infected for 2 hr with *Y. pestis* CO92 (MOI of 30∶1). After washing with PBS, cells were fixed with 10% formalin for 48 hr and subsequent staining of the bacteria and macrophages was performed as described above.

### Dual Labeling of Y. Pestis

RAW264.7 macrophages were seeded as described above in 96 well imaging plates and cells were infected with GFP expressing *Y. pestis* at an MOI of 30∶1 and incubated at 37°C and 5% CO_2_. After 2 hr, cells were washed twice with PBS and fixed for 15 minutes in 4% paraformaldehyde. After washing, cells were blocked for 1 hr, stained with αFI primary antibody (2 µg/ml in blocking buffer) and then subsequently stained with anti-mouse Alexa 568 (1∶500 dilution in blocking buffer). The cells were stained with Hoechst nuclear dye and CellMask Deep Red dye for host cell cytoplasmic staining. The cells were imaged and analyzed as described in the section –“image acquisition and object identification”. Experiments were done in six replicates and repeated at least two independent times.

### NF-κB Translocation Assay

RAW264.7 macrophages were seeded as described above. The next day, cells were pre-treated for 2 hr with 0.5% DMSO or inhibitor control wedelolactone (50 µM, Enzo BML-E1316-0001) or 20 µM of compounds from Sigma-Aldrich LOPEC^1280^ library. Appropriate wells were then treated with lipopolysaccharide (LPS) purified from *E.coli* K12 (1 µg/ml, Invivogen), Pam3CSK4 (1 µg/ml Invivogen) or infected with 10∶1 MOI of *Y. pestis* CO92 or the avirulent strain Pgm^−^, pPst^−^, as appropriate. After incubation for 30 min, cells were washed 2 times with PBS, and then fixed and permeabilized, as described for the phagocytosis assay. To detect endogenous NF-κB, cells were incubated for 1 hr with the NF-κB primary antibody (Thermo Scientific 1861301). After washing and incubation for 30 min with the anti-rabbit Dylight 488 secondary antibody (Thermo Scientific 35552), cells were stained with the Hoechst nuclear dye (Invitrogen H3570) and the CellMask Deep Red dye (Invitrogen H32721).

For dose response studies, RAW264.7 macrophages were pretreated for 2 hr with 2-fold serial dilutions of the select small molecules. Cells were infected with *Y. pestis* CO92 (MOI of 10∶1) for 30 min, washed with PBS and fixed with 10% formalin for 48 hr. Subsequent staining of the macrophages was performed as described above.

To test if inhibition of NF-κB activation by the select hit compounds was independent of the applied stimulus, RAW264.7 macrophages were pretreated for 2 hr with 10 µM of the indicated compounds and then treated with either *E. coli* K12 LPS (1 µg/ml), Pam3CSK4 (1 µg/ml) or infected with 10∶1 MOI of *Y. pestis* CO92 for 30 min. Cells were washed, stained with NF-κB antibody and imaged as described below.

### Image Acquisition and Object Identification

Automated image acquisition of the plates was performed using an Opera confocal reader (model 3842-Quadruple Excitation High Sensitivity (QEHS), Perkin Elmer). Images were acquired from multiple optical fields (6 fields) within each well using a 20× water objective in four independent channels. The images were then analyzed within the Opera environment using standard and assay customized Acapella scripts. During image analysis, the use of separate nuclear Hoechst dye and whole cell CellMask Deep Red stain enabled accurate identification and segmentation of the nuclei and the cells respectively. Using the above segmented nuclei and cytoplasmic regions, a nuclear mask and whole cell mask were created on the signal image.

For the phagocytosis assay, Acapella’s Spot Detection algorithm was used to detect bacteria and quantify internalized and cell-associated bacteria. Bacteria were detected as spots in a specified search region (WholeCell) having a higher intensity than its surroundings. To separate the spatial noise peaks and other artifacts, all spots detected initially were regarded as spot candidates. Spot candidates were then separated as “Classified Spots” based on two parameters. 1. contrast (*i.e*. contrast between the maximum intensity and the local background intensity near the spot candidate) and 2. spot-to-cell intensity (*i.e*. the ratio between the maximum intensity of the spot candidate and the average intensity of the cell to which the spot object belongs). The spot detection analyses results in various properties of spots and host cells as described in Table S1. “Classified spots”, “number of spots per cell” and “Integrated Spot Signal Per Cellular Signal local background subtracted, over the whole SearchRegion” and “cell number” parameters were primarily used for further analysis.

The internalized and cell-associated bacteria were confirmed by acquiring confocal Z stack images using the Opera High-content imager with a 40×water objective. A total of 15 images at different Z planes (separated by 1 µm) were acquired and imported to ImageJ software to reconstruct the Z stack.

For the *Y. pestis* dual labeling studies, the cells were imaged as described above and analyzed using Columbus spot analysis script. During analysis, single cell data from multiple wells were combined using R. Cells with zero associated spots were removed from the data, and the mean relative red and green spot intensities were plotted for the remaining cells.

For the NF-κB translocation assay, a nuclear translocation algorithm was applied to quantitate NF-κB signal intensity in the cytoplasm and nucleus. Based on the Hoechst DNA dye and whole cell dye CellMask Deep Red, the nuclei and cells were respectively identified and the analysis script was applied to perform the masking and segmentation operations. The optimized parameters of the nuclei and cytoplasmic detection algorithms were then applied to generate clearly segmented nuclei and cells respectively and subsequently to create a nuclear and whole-cell mask. By subtracting the nuclear region from the whole cell region (defined by Cell Mask Deep Red), a purely cytoplasmic sampling region was generated. A nuclear ring region was generated by eroding the nuclear mask. The ring was necessary due to large areas of nonstaining in the nucleolar regions. The distribution of fluorescently labeled NF-κB between the nucleus and cytoplasmic regions was then detected in the signal channel (488-image channel).

### Bacterial Viability Assay

RAW264.7 macrophages were seeded at a density of 2.5×10^5^ cells/well in 24-well plates. The next day, the cells were pretreated for 2 hr with 0.5% DMSO or the identified bioactive compounds at concentration of 10 µM (or unless otherwise noted). (0.5 ml/well). Cells were then infected with *Y*. *pestis* CO92 strain (30∶1 MOI) and incubated at 37°C and 5% CO_2_ for 2 hr. Cells were washed 3 times with PBS to remove extracellular bacteria, and either lysed with 0.1% TX-100 or further incubated for 6 hr (total 8 hr infection) in the presence of the compounds before macrophage lysis. Lysate aliquots were serially diluted and plated onto SBA plates and incubated at 28°C for 48 hr. Bacterial load was determined by the number of CFU/ml present in the sample (CFU/ml = number of colonies on the plate multiplied by the dilution factor and adjusted for a volume of 1 ml). Statistical analysis of the data was performed using one way analysis of variance and Dunnett test comparing data from test compound with DMSO control.

### In Vitro Bacterial Growth Inhibition and Viability Assays

For *in vitro* growth inhibition assay, *Y. pestis* CO92 was streaked from frozen stocks onto SBA plates and incubated at 35°C for 24 hr. Next day, bacterial colonies from the plates were resuspended in cDMEM medium, OD_600_ measured and bacterial concentration was determined. Bacterial cultures were seeded (5×10^5^ CFU/ml) in 96-well plates and treated with DMSO or select small bioactive molecules (20 µM). The plates were incubated at 37°C and 5% CO_2_ for 18 hr, and bacterial growth was determined by measuring the OD_600_.

For bacterial viability assay, bacterial cultures were seeded (5×10^5^ CFU/ml) in 96-well plates in cDMEM medium and treated with DMSO or select small bioactive molecules (20 µM) and incubated at 37°C and 5% CO2. At 2 hr and 8 hr, bacterial viability was determined using the standard colony-forming assay as described above.

### Cell Viability Assay

RAW264.7 macrophages seeded in 96-well plates were treated with the indicated concentrations of the identified hit compounds, incubated at 37°C and 5% CO_2_ and after 8 hr, 25 µl of MTT (1 mg ml^−1^) dye was added. Cells were further incubated for 2 hr and the reaction was stopped by adding an equal volume of lysis buffer (50% dimethylformamide and 20% SDS, pH 4.7) and plates were incubated overnight at 37°C. After 24 hrs absorbance was read at 570 nm in a multi-well plate reader (Tecan Safire II, San Jose, CA).

### Immunoblotting

RAW264.7 macrophages were pretreated with 0.5% DMSO or compounds (10 µM) for 2 hr and then infected with 10∶1 MOI of *Y*. *pestis* CO92 for time points of 15, 20 or 60 mins or as indicated. Cells were washed and then lysed in buffer containing 50 mM Tris-HCl (pH 7.4), 150 mM NaCl, 2 mM EDTA, 1% Triton X-100, 10 mM NaF, 25 mM β-glycerophosphate, 1 mM sodium orthovanadate, protease and phosphatase inhibitor cocktails. Cell lysates were electrophoresed using SDS-PAGE and immunoblotting was performed using IκBα (Catalog # 9242, Cell Signaling Technology Inc, Danver, MA) or p-IκBα (Ser 32, Catalog # 2859, Cell Signaling Technology Inc., Danvers, MA) antibody. The blots were also probed with mouse anti transferrin receptor antibody (Catalog # 13-6890, Life technologies) to correct for protein loading. Immunoreactive proteins were visualized by enhanced chemiluminescence. The pIκBα and transferrin receptor band intensities were quantified as pixels using Genetools version 4.0 from SynGene (Cambridge, England) software.

### Statistical Analysis

For the screening data, statistical analysis was performed using R [51]. Each measurement was plate-level normalized using a robust Z-score. The median value for infected wells on each plate was subtracted from the well value, which was then divided by the scaled median absolute deviation (a robust estimate of the standard deviation). Normalized values were visually examined for spatial artifacts on each plate using the R/Bioconductor package *prada*. Principal components analysis was performed using R. The medoid infected DMSO-treated control well was determined using the R package *cluster* and the Mahalanobis distance from each well to the medoid was determined in principal components space.

## Supporting Information

Figure S1 **Optimization of the phagocytosis assay.** RAW264.7 macrophages were infected with different MOIs (10∶1, 30∶1 and 50∶1) of avirulent strain *Y. pestis* (Pgm^−^,pPst^−^) and for different time points (1, 2 and 4 hr), and then stained with αF1 antibody. The number of internalized bacteria was enumerated using spot analysis software.
(PDF)

Figure S2 **Optimization of the** ***Y*** **.** ***pestis*** **induced NF-κB translocation assay.** (**A**) Quantitation of NF-κB translocation in RAW264.7 macrophages treated with chemical inducers LPS (1 µg/ml) or Pam3CSK4 (1 µg/ml) or infected with 10∶1 MOI of the avirulent strain of *Y*. *pestis* (Pgm^−^,pPst^−^). After 30 min or 1 h, cells were washed, fixed, permeabilized, stained with αNF-κB antibody and images were acquired and analyzed. (**B**) Quantitation of NF-κB translocation in RAW264.7 macrophages infected with 10∶1, 30∶1 or 50∶1 MOI of the avirulent strain of *Y*. *pestis* (Pgm^−^,pPst^−^). After 30 min, the cells were washed, fixed, permeabilized, stained with αNF-κB antibody and acquired images were analyzed.
(PDF)

Figure S3 **Temporal oscillation patterns of NF-κB activation and inhibition.** RAW264.7 macrophages were either left untreated or treated with LPS (1 µg/ml) or infected with 10∶1 MOI of *Y*. *pestis* CO92. After 0.5, 1, 2 or 3 hr, cells were washed fixed, permebailized, stained with αNF-κB antibody and acquired images were analyzed.
(PDF)

Figure S4 **Identified hits do not** ***in vitro*** **inhibit bacterial growth.** *Y. pesits* CO92 (5×10^5^ CFU/ml) was treated with (1) DMSO control (0.5%) or 20 µM of compounds, (2) rac-2-Ethoxy-3 octadecanamido-1-propylphosphocholine, (3) parthenolide, (4) wortmannin and (5) tyrphostin A9. After 18 hr absorbance at 600 nm was measured.
(PDF)

Figure S5 **Identified hits do not** ***in vitro*** **inhibit bacterial viability.** *Y. pestis* CO92 was incubated with (1) DMSO control (0.5%) or 20 µM of compounds, (2) rac-2-Ethoxy-3 octadecanamido-1-propylphosphocholine, (3) parthenolide, (4) wortmannin and (5) tyrphostin A9. After 2 hr and 8 hr bacterial viability was determined using colony forming assay.
(PDF)

Figure S6 **Cell-based cytotoxicity assay.** RAW264.7 macrophages were treated with indicated concentrations of select hit compounds and after 8 hr cell viability was measured using an MTT assay.
(PDF)

Figure S7 **Detection of pCD1 plasmid.** Nucleic acid extracted from bacteria grown on SBA plates incubated at either 28°C or 35°C for 24 hr as well as from *Y. pestis* infected macrophages 2 hr post infection, was subjected to PCR analysis using the *Y. pestis* PCR primer set (BEI resources, Catalog No. NR-9688). Linearized plasmid DNA (BEI Resources, Catalog # NR-9551) was used as an internal control DNA for *Y. pestis* plasmid detection. (A) Agarose gel showing the presence of 1.9 kb pCD1 plasmid in *Y. pestis* grown at 28°C or 35°C on SBA plates. Lane 1, High molecular weight DNA maker; Lane 2, internal control *Y. pestis* DNA showing ∼130 bp pCD1 amplicon; Lane 3, water negative control; Lane 4, Bacteria grown at 28°C showing the 1.9 kb pCD1 amplified product; Lane 5, Bacteria grown at 35°C showing the 1.9 kb pCD1 amplified product; Lane 6, low molecular DNA marker. (B) Agarose gel showing the presence of pCD1 plasmid (197 bp PCR product) in bacteria grown at 28°C (Lane 2) or 35°C (lane 3) on SBA plates or from RAW264.7 macrophages infected for 2 hrs with *Y. pestis* CO92 grown at 28°C (lane 4) or 35°C (lane 5). Lane 1, Low molecular weight DNA marker. The PCR primers used for amplification are- Forward primer 5′ GGCAGTAGACCAGGAATGGA 3′ and Reverse primer 5′ TGAGTGAGCGTAACGACTGG 3′.
(PDF)

Table S1 **List of the image output features that were collected during image analysis for the phagocytosis assay and their related parametric description.**
(PDF)

Table S2 **List of the image output features that were collected during image analysis for the NF-κB translocation assay and their related parametric description.**
(PDF)

Table S3 **Real time Taqman PCR assay targets and bacterial growth conditions.**
(PDF)

Movie S1 **Z-stack confocal images of macrophages infected with** ***Y. pestis*** **CO92.** Macrophages were infected with 30∶1 MOI of *Y. pestis* CO92 for 2 hr, washed, fixed and stained with αF1 antibody to detect the bacteria. A total of 15 images were collected and the distance between the planes is 1 µm. *Y. pestis* was pseudo colored green, while cell mask deep red staining the macrophages were pseudo colored Indian red.
(ZIP)
