# Supplementary material for: Integrating High-Content Imaging and Chemical Genetics to Probe Host Cellular Pathways Critical for Yersinia Pestis Infection
Source: PLoS One. 2013 Jan 30;8(1):e55167. doi: 10.1371/journal.pone.0055167 (PMC3559335; doi:10.1371/journal.pone.0055167)
Supplement: Figure S1 — Optimization of the phagocytosis assay. RAW264.7 macrophages were infected with different MOIs (10∶1, 30∶1 and 50∶1) of avirulent strain Y. pestis (Pgm−,pPst−) and for different time points (1, 2 and 4 hr), and then stained with αF1 antibody. The number of internalized bacteria was enumerated using spot analysis software. (PDF) [file pone.0055167.s001.pdf]

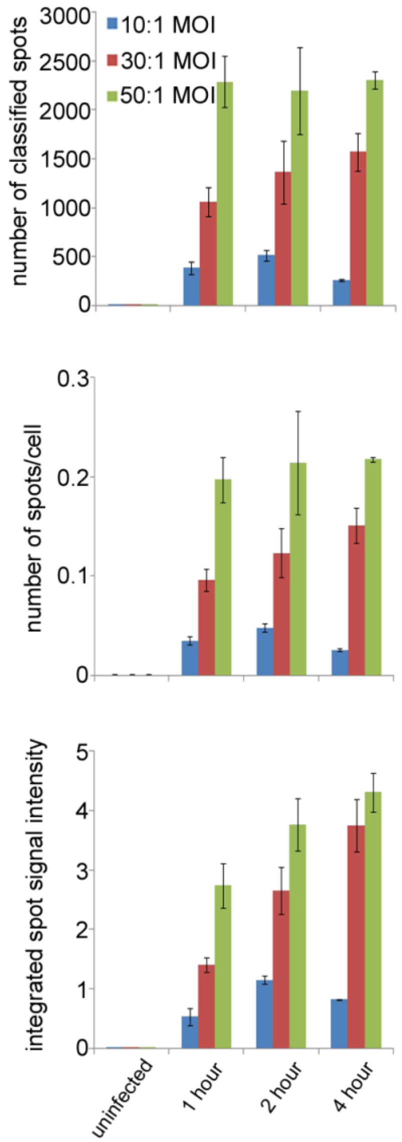

Figure S1. Optimization of the phagocytosis assay. RAW264.7 macrophages were infected with different MOIs (10:1, 30:1 and 50:1) of avirulent strain *Y. pestis* (Pgm^-,$pPst^-$) and for different time points (1, 2 and 4 hr), and then stained with αF1 antibody. The number of internalized bacteria was enumerated using spot analysis software.
