# Supplementary material for: Integrating High-Content Imaging and Chemical Genetics to Probe Host Cellular Pathways Critical for Yersinia Pestis Infection
Source: PLoS One. 2013 Jan 30;8(1):e55167. doi: 10.1371/journal.pone.0055167 (PMC3559335; doi:10.1371/journal.pone.0055167)
Supplement: Figure S2 — Optimization of the Y . pestis induced NF-κB translocation assay. (A) Quantitation of NF-κB translocation in RAW264.7 macrophages treated with chemical inducers LPS (1 µg/ml) or Pam3CSK4 (1 µg/ml) or infected with 10∶1 MOI of the avirulent strain of Y. pestis (Pgm−,pPst−). After 30 min or 1 h, cells were washed, fixed, permeabilized, stained with αNF-κB antibody and images were acquired and analyzed. (B) Quantitation of NF-κB translocation in RAW264.7 macrophages infected with 10∶1, 30∶1 or 50∶1 MOI of the avirulent strain of Y. pestis (Pgm−,pPst−). After 30 min, the cells were washed, fixed, permeabilized, stained with αNF-κB antibody and acquired images were analyzed. (PDF) [file pone.0055167.s002.pdf]

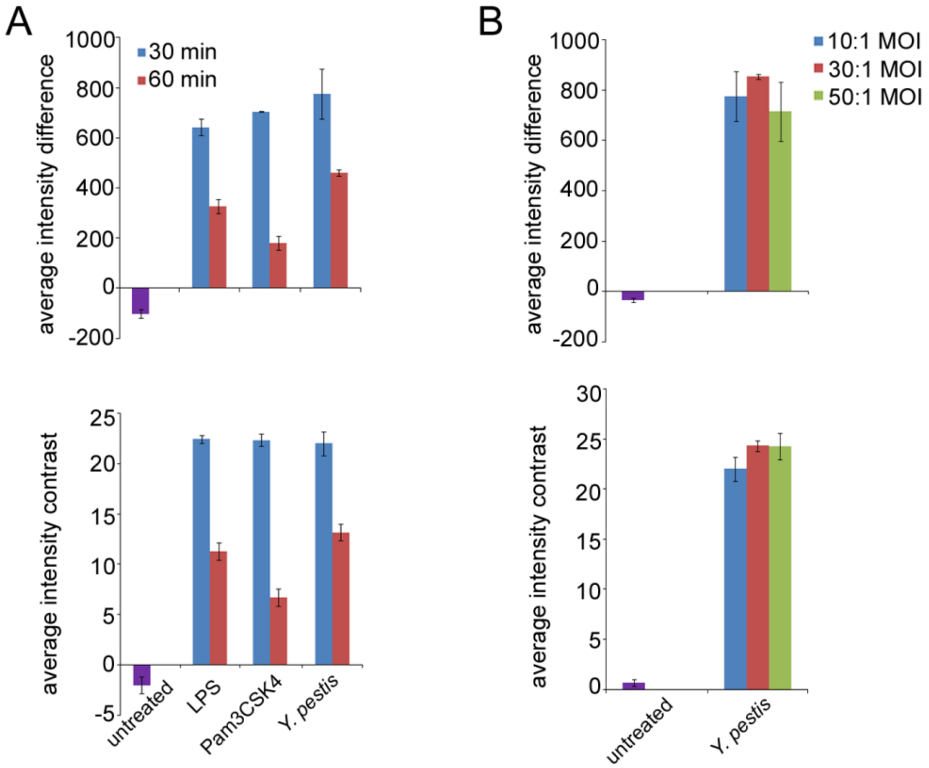

Figure S2. Optimization of the *Y*. *pestis* induced NF-κB translocation assay. (**A**) Quantitation of NF-κB translocation in RAW264.7 macrophages treated with chemical inducers LPS (1 µg/ml) or Pam3CSK4 (1 µg/ml) or infected with 10:1 MOI of the avirulent strain of *Y*. *pestis* (Pgm^-,$pPst^-$). After 30 min or 1 h, cells were washed, fixed, permeabilized, stained with αNF-κB antibody and images were acquired and analyzed. (**B**) Quantitation of NF-κB translocation in RAW264.7 macrophages infected with 10:1, 30:1 or 50:1 MOI of the avirulent strain of *Y*. *pestis* (Pgm^-,$pPst^-$). After 30 min, the cells were washed, fixed, permeabilized, stained with α NF-κB antibody and acquired images were analyzed.
