# Supplementary material for: Integrating High-Content Imaging and Chemical Genetics to Probe Host Cellular Pathways Critical for Yersinia Pestis Infection
Source: PLoS One. 2013 Jan 30;8(1):e55167. doi: 10.1371/journal.pone.0055167 (PMC3559335; doi:10.1371/journal.pone.0055167)
Supplement: Figure S3 — Temporal oscillation patterns of NF-κB activation and inhibition. RAW264.7 macrophages were either left untreated or treated with LPS (1 µg/ml) or infected with 10∶1 MOI of Y. pestis CO92. After 0.5, 1, 2 or 3 hr, cells were washed fixed, permebailized, stained with αNF-κB antibody and acquired images were analyzed. (PDF) [file pone.0055167.s003.pdf]

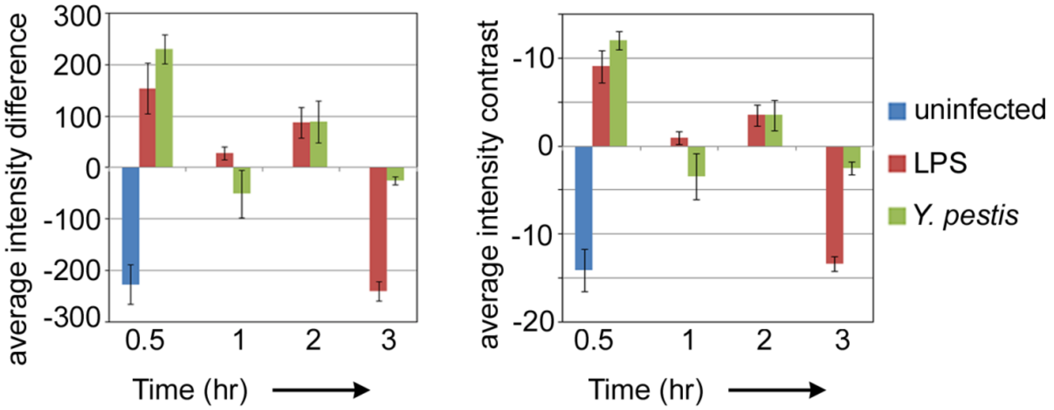

Figure S3. Temporal oscillation patterns of NF-κB activation and inhibition. RAW264.7 macrophages were either left untreated or treated with LPS (1 µg/ml) or infected with 10:1 MOI of *Y*. *pestis* CO92. After 0.5, 1, 2 or 3 hr, cells were washed fixed, permeabilized, stained with α NF-κB antibody and acquired images were analyzed.
