# Supplementary material for: Integrating High-Content Imaging and Chemical Genetics to Probe Host Cellular Pathways Critical for Yersinia Pestis Infection
Source: PLoS One. 2013 Jan 30;8(1):e55167. doi: 10.1371/journal.pone.0055167 (PMC3559335; doi:10.1371/journal.pone.0055167)
Supplement: Figure S4 — Identified hits do not in vitro inhibit bacterial growth. Y. pesits CO92 (5×10^5^ CFU/ml) was treated with (1) DMSO control (0.5%) or 20 µM of compounds, (2) rac-2-Ethoxy-3 octadecanamido-1-propylphosphocholine, (3) parthenolide, (4) wortmannin and (5) tyrphostin A9. After 18 hr absorbance at 600 nm was measured. (PDF) [file pone.0055167.s004.pdf]

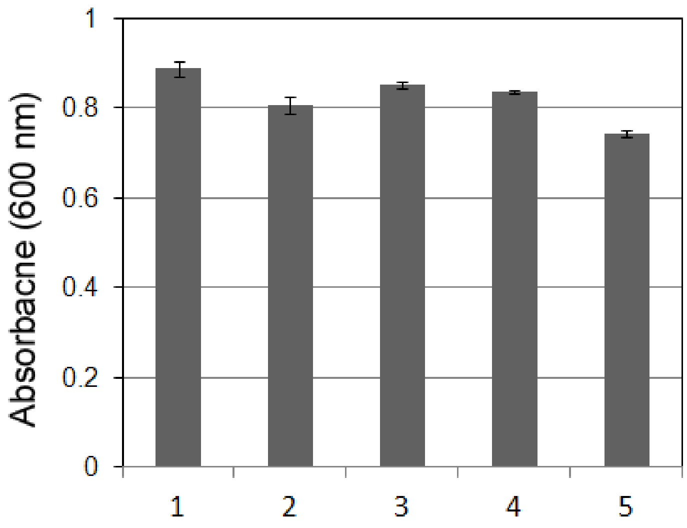

Figure S4. Identified hits do not *in vitro* inhibit bacterial growth. *Y. pestis* CO92 (5 x10^5 CFU/ml) was treated with (1) DMSO control (0.5%) or 20 µM of compounds, (2) rac-2-Ethoxy-3 octadecanamido-1-propylphosphocholine, (3) parthenolide, (4) wortmannin and (5) tyrphostin A9. After 18 hr absorbance at 600 nm was measured.
