# Supplementary material for: Integrating High-Content Imaging and Chemical Genetics to Probe Host Cellular Pathways Critical for Yersinia Pestis Infection
Source: PLoS One. 2013 Jan 30;8(1):e55167. doi: 10.1371/journal.pone.0055167 (PMC3559335; doi:10.1371/journal.pone.0055167)
Supplement: Figure S6 — Cell-based cytotoxicity assay. RAW264.7 macrophages were treated with indicated concentrations of select hit compounds and after 8 hr cell viability was measured using an MTT assay. (PDF) [file pone.0055167.s006.pdf]

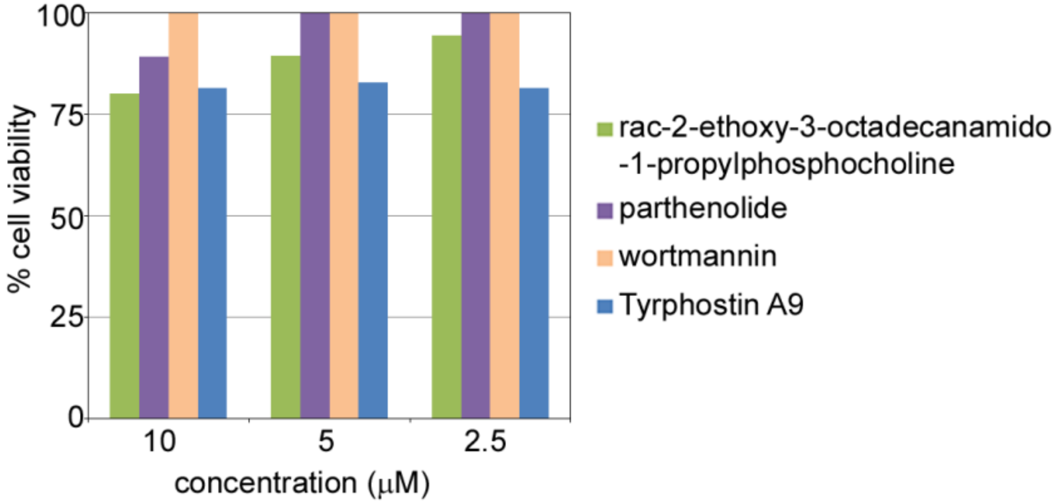

Figure S6. Cell-based cytotoxicty assay. RAW264.7 macrophages were treated with indicated concentrations of select hit compounds and after 8 hr cell viability was measured using an MTT assay.
