# Supplementary material for: Integrating High-Content Imaging and Chemical Genetics to Probe Host Cellular Pathways Critical for Yersinia Pestis Infection
Source: PLoS One. 2013 Jan 30;8(1):e55167. doi: 10.1371/journal.pone.0055167 (PMC3559335; doi:10.1371/journal.pone.0055167)
Supplement: Figure S7 — Detection of pCD1 plasmid. Nucleic acid extracted from bacteria grown on SBA plates incubated at either 28°C or 35°C for 24 hr as well as from Y. pestis infected macrophages 2 hr post infection, was subjected to PCR analysis using the Y. pestis PCR primer set (BEI resources, Catalog No. NR-9688). Linearized plasmid DNA (BEI Resources, Catalog # NR-9551) was used as an internal control DNA for Y. pestis plasmid detection. (A) Agarose gel showing the presence of 1.9 kb pCD1 plasmid in Y. pestis grown at 28°C or 35°C on SBA plates. Lane 1, High molecular weight DNA maker; Lane 2, internal control Y. pestis DNA showing ∼130 bp pCD1 amplicon; Lane 3, water negative control; Lane 4, Bacteria grown at 28°C showing the 1.9 kb pCD1 amplified product; Lane 5, Bacteria grown at 35°C showing the 1.9 kb pCD1 amplified product; Lane 6, low molecular DNA marker. (B) Agarose gel showing the presence of pCD1 plasmid (197 bp PCR product) in bacteria grown at 28°C (Lane 2) or 35°C (lane 3) on SBA plates or from RAW264.7 macrophages infected for 2 hrs with Y. pestis CO92 grown at 28°C (lane 4) or 35°C (lane 5). Lane 1, Low molecular weight DNA marker. The PCR primers used for amplification are- Forward primer 5′ GGCAGTAGACCAGGAATGGA 3′ and Reverse primer 5′ TGAGTGAGCGTAACGACTGG 3′. (PDF) [file pone.0055167.s007.pdf]

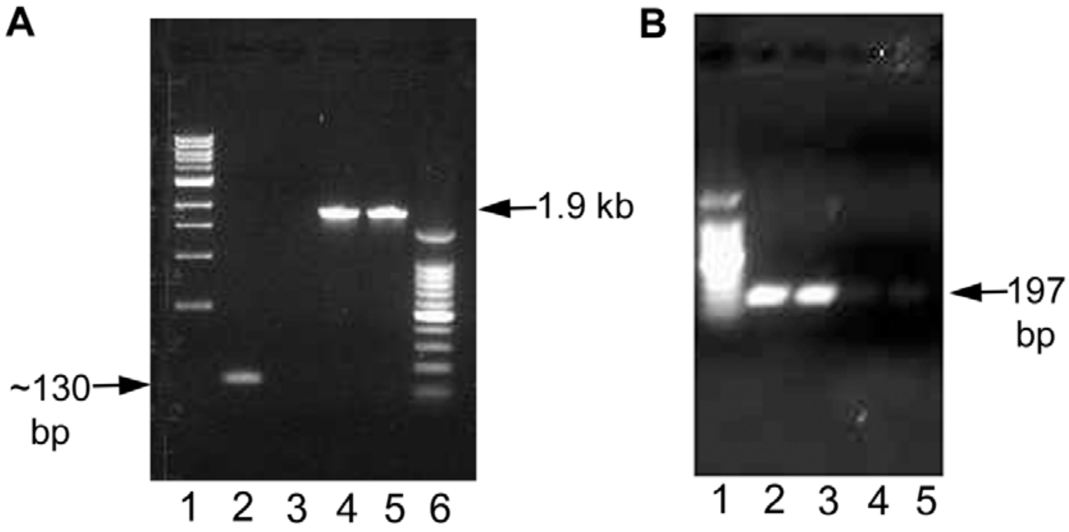

Figure S7. Detection of pCD1 plasmid. Nucleic acid extracted from bacteria grown on SBA plates incubated at either 28°C or 35°C for 24 hr as well as from *Y. pestis* macrophages 2 hr post infection, was subjected to PCR analysis using the *Y. pestis* PCR primer set (BEI resources, Catalog No. NR-9688). Linearized plasmid DNA (BEI Resources, Catalog # NR-9551) was used as an internal control DNA for *Y. pestis* plasmid detection. (A) Agarose gel showing the presence of 1.9 kb pCD1 plasmid in *Y. pestis* grown at 28°C or 35°C on SBA plates. Lane 1, High molecular weight DNA maker; Lane 2, internal control *Y. pestis* DNA showing ~130 bp pCD1 amplicon; Lane 3, water negative control; Lane 4, Bacteria grown at 28°C showing the 1.9 kb pCD1 amplified product; Lane 5, Bacteria grown at 35°C showing the 1.9 kb pCD1 amplified product; Lane 6, low molecular DNA marker. (B) Agarose gel showing the presence of pCD1 plasmid (197 bp PCR product) in bacteria grown at 28°C (Lane 2) or 35°C (lane 3) on SBA plates or from RAW264.7 macrophages infected for 2 hrs with *Y. pestis* CO92 grown at 28°C (lane 4) or 35°C (lane 5). Lane 1, Low molecular weight DNA marker. The PCR primers used for amplification are- Forward primer 5' GGCAGTAGACCAGGAATGGA 3' and Reverse primer 5' TGAGTGAGCGTAACGACTGG 3'.
