# Supplementary material for: Integrating High-Content Imaging and Chemical Genetics to Probe Host Cellular Pathways Critical for Yersinia Pestis Infection
Source: PLoS One. 2013 Jan 30;8(1):e55167. doi: 10.1371/journal.pone.0055167 (PMC3559335; doi:10.1371/journal.pone.0055167)
Supplement: Table S1 — List of the image output features that were collected during image analysis for the phagocytosis assay and their related parametric description. (PDF) [file pone.0055167.s008.pdf]

Table S1. List of the image output features that were collected during image analysis for the phagocytosis assay and their related parametric description.

Image output parameters	Parametric description
Cell number	Total number of cells (macrophages) in the image fields
Number of spot (bacteria) candidates	The number of local intensity maxima found within the search region
Number of classified spots (internalized bacteria)	All spot candidates with attribute values for Contrast and Spot To Cell intensity above the limits set by the input parameters (SpotMinimumContrast and SpotMinimumToCellIntensity)
Number of spots per cell (per object)	number of spots (bacteria)/cell (macrophage)
Number of spots per cell area (per object area), unit spots per pixel.	The number of detected spots per visible cell area in unit spots per pixel. the number spots is normalized for the area of the SearchRegion.
Integrated Spot Signal Per Cellular Signal, over the whole SearchRegion.	The Integrated pixel intensities for all spots normalized to the integrated pixel intensities for all cells inside the SearchRegion)
Integrated Spot Signal Per Cellular Signal local background subtracted, over the whole SearchRegion.	The integrated pixel intensity local backfround subtracted from all spots normalized to integrated pixel intensity over all cells.
Integrated spot signal per SearchRegion area (per object area), unit counts per pixel.	The integrated pixel intensities for all spots normalized to the visible cell area
Integrated spot signal local backgroundSubtracted per SearchRegion area (per object area), unit counts per pixel.	The integrated pixel intensities for the local background subtracted from all spots normalized to the visible cell area (per area of (SearchRegion). Unit counts per pixel..
