# Supplementary material for: Integrating High-Content Imaging and Chemical Genetics to Probe Host Cellular Pathways Critical for Yersinia Pestis Infection
Source: PLoS One. 2013 Jan 30;8(1):e55167. doi: 10.1371/journal.pone.0055167 (PMC3559335; doi:10.1371/journal.pone.0055167)
Supplement: Table S2 — List of the image output features that were collected during image analysis for the NF-κB translocation assay and their related parametric description. (PDF) [file pone.0055167.s009.pdf]

Table S2. List of the image output features that were collected during image analysis for the NF-κB translocation assay and their related parametric description.

Image output parameters	Parametric description
Cell number	Total number of cells (macrophages) in all the image fields
Average nucleus size	Cell nuclear size
Average Intensity of Nuclei	Signal channel intensity in the nuclei
Average Intensity of Cytoplasm	Signal channel intensity in whole cells
Average Normalized Nucleus Intensity	Nucleus Fraction (nucleus intensity/total intensity) of signal channel intensity
Average Normalized Cytoplasm Intensity	Cytoplasm Fraction (cytoplasm intensity/total intensity) of signal channel intensity
Average Intensity Ratio	Nuclear intensity/cytoplasm intensity (signal channel)
Average Intensity Contrast	(NucIntensity-CytoIntensity/Nuc+Cyto)*100
Average Intensity Difference	NucIntensity-CytoIntensity
