# Supplementary material for: Integrating High-Content Imaging and Chemical Genetics to Probe Host Cellular Pathways Critical for Yersinia Pestis Infection
Source: PLoS One. 2013 Jan 30;8(1):e55167. doi: 10.1371/journal.pone.0055167 (PMC3559335; doi:10.1371/journal.pone.0055167)
Supplement: Table S3 — Real time Taqman PCR assay targets and bacterial growth conditions. (PDF) [file pone.0055167.s010.pdf]

Table S3. Real time Taqman PCR assay targets and bacterial growth conditions.

Plasmids or gene targets	Bacterial Growth condition temperatures	[g]CT value
[a]PTC-CAF MM	[f]NA	33.08
[b]NTC	NA	0
[c]Yp	28°C	14.52
[d]Yp-RAW	28°C	22.57
Yp	35°C	14.52
Yp-RAW	35°C	23.98
[e]PTC-PIM MM	NA	30.20
[b]NTC	NA	0
Yp	28°C	11.06
Yp-RAW	28°C	22.58
Yp	35°C	11.85
Yp-RAW	35°C	23.70
[c]PTC-PLA MM	NA	29.21
[b]NTC	NA	0
Yp	28°C	10.31
Yp-RAW	28°C	20.80
Yp	35°C	10.88
Yp-RAW	35°C	21.41

[a] Positive template control pFra plasmid master mix, [b] no template control,[c] Yp; *Y. pestis* CO92, [d] Yp infected RAW macrophages [e]Positive template control pPCP1plasmid master mix, [f] not applicable, and [g] cycle threshold.
